# Self-assembled supramolecular structures of *O*,*N*,*N*′ tridentate imidazole–phenol Schiff base compounds†

**DOI:** 10.1039/c9ra10488g

**Published:** 2020-02-24

**Authors:** Kristy-Lyn Barry, Craig D. Grimmer, Orde Q. Munro, Matthew P. Akerman

**Affiliations:** School of Chemistry and Physics, University of KwaZulu-Natal Private Bag X01, Scottsville Pietermaritzburg 3209 South Africa akermanm@ukzn.ac.za; Molecular Sciences Institute, School of Chemistry, WITS University Johannesburg South Africa

## Abstract

Three imidazole-derived Schiff base compounds comprising an *N*-methyl imidazole group coupled to a phenol ring through an imine bond were synthesised. The structures differ by the substituent on the phenol ring at the 4-position: methyl (1), *tert*-butyl (2) and hydrogen (3). The compounds were synthesised using both a traditional reflux in solvent as well as an environmentally friendly solid-state reaction. Compounds (1)–(3) as well as the hemihydrate of (3) were all studied by single crystal X-ray diffraction. The asymmetric unit of compound (1) consists of two nominally planar molecules linked by hydrogen bonds to form a dimeric supramolecular structure. This dimeric structure was ubiquitous for the anhydrous forms of (1)–(3). The complementary hydrogen bonding motif between the imidazole N atoms and the phenol OH results in a stable 16-membered hydrogen-bonded ring. The asymmetric unit of (3) comprises two symmetry-independent molecules one of which has co-planar imidazole and phenol rings while the other shows a significantly oblique orientation. The hemihydrate of (3) similarly forms extensive hydrogen bonds, though in the form of a water-bridged dimeric structure. The hydrogen bond lengths (D⋯A) for compounds (1)–(3) are relatively short, ranging from 2.662(1) to 2.688(1) Å. DFT was used to understand the relative stability of the monomeric and dimeric species. These showed the hydrogen-bonded supramolecular structures were *ca.* 101 kJ mol^−1^ lower in energy than the non-interacting monomers. Scan simulations were used to calculate the total energy of the molecule as a function of phenyl ring rotation and showed why the expected planar configuration for a conjugated π-system was not observed experimentally. The barrier to rotation was found to be relatively low, 7.97(6) kJ mol^−1^, with the lowest energy conformations subtending dihedral angles of 22.319, 24.265 and 25.319° for molecules (1), (2) and (3), respectively. The electrostatic potential maps are able to succinctly explain the stability of the hydrogen bonds through the partial charges of the interacting atoms. TD-DFT simulations and analysis of the simulated and experimental UV/visible spectra suggest that the dimeric supramolecular structure is a stable species in solution. This was confirmed through ^1^H NMR titrations and an equilibrium constant of 0.16(5) M^−1^ was estimated.

## Abstract 2

Three imidazole-derived Schiff base compounds comprising an *N*-methyl imidazole group coupled to a phenol ring through an imine bond were synthesised. The solid state and solution state supramolecular structures as well as energetics are explored.

## Graphical abstract

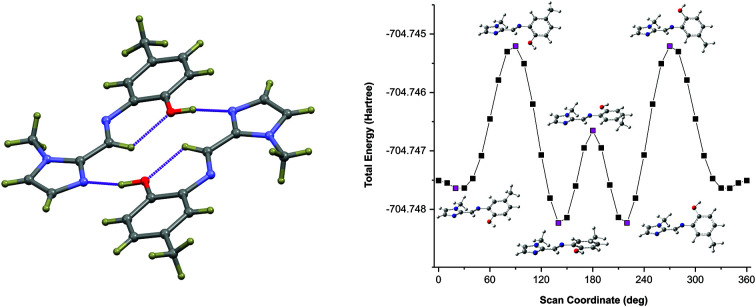

## 1. Introduction

Imidazole-derived Schiff bases are readily synthesised *via* a condensation reaction between the corresponding imidazole–carboxaldehyde and amine derivative. In general, aromatic amine and aromatic aldehyde precursors result in higher yields than Schiff bases synthesised from aliphatic precursors. This is attributed to the electron dense aryl groups stabilising the imine bond through electron delocalisation.^1^ This ease of synthesis coupled with the ubiquitous nature of imidazole moieties in enzymes, proteins and pharmaceuticals render it a favourable choice in biological studies.^1,2^ Additionally, Schiff base ligands are able to coordinate and stabilise metals in a wide range of oxidation states.^2–8^ Metal complexes of imidazole-based Schiff base ligands have therefore found application in a wide variety of fields including medicinal and biological chemistry as well as catalysis.^1^

**Scheme 1:**
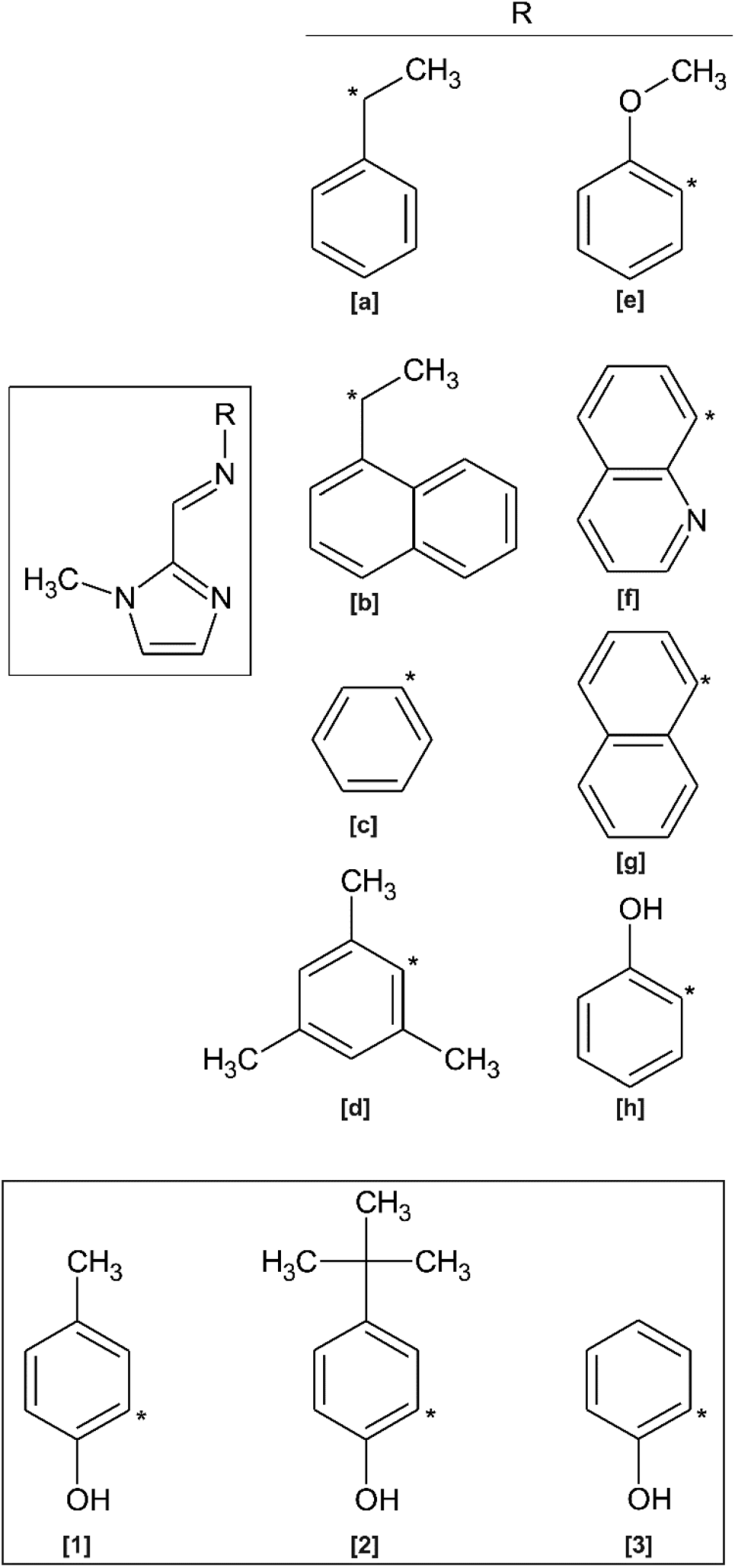
[Main] Structure of previously reported imidazole-based Schiff base ligands related to the present study. [Inset] Structures of compounds (1)–(3) reported herein.

In recent years, derivatives of the 1-methyl-imidazol-2-yl methylidine Schiff Base ligand have been co-ordinated to various metals for a wide variety of applications. In contrast to imidazole ligands, the *N*-methyl group prevents ionisation upon metal ion coordination, ensuring the ligand remains neutral.^9^ A search of the Cambridge Structural Database (CSD) shows that no free ligand crystal structures containing the 1-methyl-imidazol-2-yl methylidine amino moiety have been reported. However, crystal structures of metal complexes containing derivatives of this chelating ligand are known (Scheme 1).^10–20^ Homochiral Fe(ii) molecular magnet complexes containing *N*,*N*′ bidentate ligands of type (a) and (b) (as shown in Scheme 1) have been synthesised, exhibit spin-crossover properties,^10–13^ and may potentially find application as a host for racemic separation.^12^ These *N*-methyl imidazole Schiff base ligands have also been used in catalysis. Rh(iii) and Ir(iii) complexes of ligand (a) and Ir(iii) complexes of ligand (b) are catalysts for the Diels–Alder reaction between methacrolein and cyclopentadiene.^14^ Rh(i) complexes of ligand (c) have also been used as catalysts for the mono and dihydroalkoxylation of alkynes^15^ and an Ir(iii) complex containing ligand (d) has been found to be an active *in situ* catalyst for indole synthesis *via* intramolecular hydroamination.^16,17^ Boudier and co-workers prepared Ni(ii) complexes of the O,*N*,*N*′ tridentate ligand (e) for use as transition metal pre-catalysts in ethylene oligomerisation.^18^ In biological applications, the *N*,*N*′,*N*′′ tridentate ligand (f) has been coordinated to Cu(ii) for purposes of creating novel anticancer chemotherapeutic agents,^19^ while Ir(iii) and Rh(iii) fluorescent complexes of ligand (g) can be used as bioimaging probes.^2^ Imidazole Schiff bases have also been used in enzyme modelling, such as the use of a trinuclear manganese complex containing ligand (h).^20^

A few N–H imidazole derivatives of ligand (h) have also been reported as free ligands and coordinated to various metal ions including, but not limited to, Fe(iii), Ni(ii) and Cd(ii).^1,21^ Mössbauer and magnetic properties of these *O*,*N*,*N*′ tridentate Fe(iii) complexes have been reported.^21^ Solid state structures of the related free ligand structures derived from 2-aminophenol and imidazole-, pyrrole- and pyridine-2-carboxaldehyde have been also reported.^22–25^ With only metal complexes of ligands (e) and (h) previously reported,^18,20^ literature related to the solid state structures of tridentate *O*,*N*,*N*′-1-methyl-2-imidazole–phenol Schiff base molecules is scarce. There are no free ligand structures reported for this class of compound.

Herein, the synthesis of three *O*,*N*,*N*′ tridentate *N*-methyl imidazole Schiff base ligands are reported (Scheme 1). Ligands (1) and (2) are novel. Ligand (3) has been previously synthesised by Kloskowski and co-workers,^20^ but has been minimally characterised. In this paper, the X-ray crystal structures of molecules (1)–(3) are reported and discussed. Density functional theory (DFT) simulations are used to further understand the solid-state structures of the ligands and their preference for forming dimeric supramolecular structures. TD-DFT is used to deconvolute the electronic properties of the ligands and speculate their solution-state configurations. Solution state dimerisation is studied through ^1^H NMR spectroscopy.

## 2. Experimental

### 2.1 Materials and methods

The starting materials 1-methyl-2-imidazolecarboxaldehyde, 2-amino-4-*tert*-butylphenol, 2-amino-4-methylphenol and 2-aminophenol were purchased from Sigma-Aldrich (Germany) and used as received. Ethanol absolute (AR grade) and toluene (LAB grade) were purchased from Merck (South Africa) and used as received.

NMR spectra for ligands (1) and (2) were recorded with a Bruker Avance III 400 MHz spectrometer equipped with a Bruker magnet (9.4 T) at frequencies of 400 MHz for ^1^H and 100 MHz for ^13^C. NMR spectra for ligand (3) were recorded with a 500 MHz Varian Unity Inova spectrometer equipped with an Oxford magnet (11.7 T) at frequencies of 500 MHz for ^1^H and 125 MHz for ^13^C. The spectra were recorded at 30 °C. All NMR spectra were processed through Topspin 3.2, patch level 7.^26^ The proton and carbon shifts were calibrated to the residual DMSO solvent signal at 2.50 ppm for ^1^H and 39.51 ppm for ^13^C. The CDCl_3_ used for the NMR spectroscopic titration was dried over CaH_2_ for 24 hours prior to use. FTIR spectra were recorded using a Bruker Alpha FTIR spectrometer equipped with an ATR platinum Diamond 1 reflectance accessory. Data were collected using 36 scans with a resolution of 1 cm^−1^. Electronic spectra were recorded from 700–200 nm using a PerkinElmer Lambda 25 double beam spectrometer (1.0 cm path length sample cell) equipped with a PerkinElmer PTP-1 Peltier temperature controller set to 25.0 °C. Elemental analysis data were collected using a Thermo Scientific Flash 2000 Organic Elemental CHNS–O Analyser. High Resolution mass spectra were acquired with a Waters Micromass LCT Premier time-of-flight mass spectrometer using electrospray ionisation in positive mode.

X-Ray data were recorded on a Bruker Apex Duo diffractometer equipped with an Oxford Instruments Cryojet operating at 100(2) K and an Incoatec microsource operating at 30 W power. Crystal and structure refinement data are given in Table 1. Selected bond lengths and angles are presented in Table 2. The data were collected with Mo Kα (*λ* = 0.71073 Å) radiation using omega and phi scans with exposures taken at 30 W X-ray power and 0.50° frame widths using APEX2.^27^ The data were reduced with the programme SAINT^27^ using outlier rejection, scan speed scaling as well as standard Lorentz and polarisation correction factors. A SADABS semi-empirical multi-scan absorption correction was applied to the data. Direct methods, SHELX-2016 (ref. 28) and WinGX,^29^ were used to solve all structures. All non-hydrogen atoms were located in the difference density map and refined anisotropically. All hydrogen atoms were treated with the standard riding model in SHELX-2016 with C–H_aromatic_ distances of 0.93 Å and *U*_iso_ = 1.2 Ueq and C–H_methyl_ distances of 0.98 Å and *U*_iso_ = 1.5 Ueq. The O–H atoms were located in the density map and allowed to refine isotropically.

**Table 1:** Crystal data and structure refinement details for (1), (2), (3) and (3)·0.5H_2_O

	(1)	(2)	(3)	(3)·0.5H_2_O
**Crystal data**				
Chemical formula	C_24_H_26_N_6_O_2_	C_15_H_19_N_3_O	C_22_H_22_N_6_O_2_	2(C_11_H_11_N_3_O)·H_2_O
Molar mass (g mol^−1^)	430.51	257.33	402.46	420.46
Crystal system, space group	Monoclinic, *P*2_1_/*n*	Monoclinic, *C*2/*c*	Monoclinic, *P*2_1_/*c*	Monoclinic, *C*2/*c*
Temperature (K)	100	100	100	100
*a*, *b*, *c* (Å)	12.849(5), 10.609(5), 16.349 (5)	36.239(6), 7.1570(12), 11.2151(19)	11.5123(13), 9.0527(8), 19.5365(18)	22.5522(16), 7.1022(5), 13.4620(9)
*α*, *β*, *γ* (°)	90, 96.668(5), 90	90, 104.478(3), 90	90, 96.232(4), 90	90, 110.400(3), 90
*V* (Å^3^)	2213.5(15)	2816.4(8)	2024.0(3)	2021.0(2)
*Z*	4	8	4	4
Radiation type	Mo Kα	Mo Kα	Mo Kα	Mo Kα
*μ* (mm^−1^)	0.09	0.08	0.09	0.10
Crystal size (mm)	0.30 × 0.12 × 0.09	0.41 × 0.23 × 0.09	0.38 × 0.19 × 0.12	0.31 × 0.16 × 0.11

**Data collection**				
Diffractometer	Bruker APEX-II CCD			
Absorption correction	Multi-scan, SADABS			
No. of measured, independent and observed [*I* > 2*σ*(*I*)] reflections	22 148, 5907, 4450	29 656, 3453, 2952	19 119, 5330, 4562	9162, 2651, 2384
*R*_int_	0.026	0.041	0.026	0.021

**Refinement**				
*R*[*F*^2^ > 2*σ*(*F*^2^)], w*R*(*F*^2^), *S*	0.047, 0.144, 1.03	0.053, 0.139, 1.11	0.041, 0.109, 1.05	0.042, 0.116, 1.06
No. of reflections	5907	3453	5330	2651
No. of parameters	301	180	279	150
H-atom treatment	H atoms treated by a mixture of independent and constrained refinement			
Δ*ρ*_max_, Δ*ρ*_min_ (e Å^−3^)	0.42, −0.23	0.40, −0.25	0.33, −0.22	0.39, −0.20

**Table 2:** Selected bond lengths and angles for compounds (1)–(3) and (3)·0.5H_2_O

Molecule	(1)^a^	(2)	(3a)	(3b)	(3)·0.5H_2_O
**Bond lengths (Å)**					
N1–C1	1.364(2)	1.372(2)	1.374(2)	1.375(2)	1.369(2)
N1–C4	1.334(2)	1.341(2)	1.341(2)	1.340(2)	1.336(2)
C1–C2	1.362(3)	1.364(2)	1.367(2)	1.368(2)	1.370(2)
C2–N2	1.368(2)	1.373(2)	1.372(2)	1.374(2)	1.371(2)
N2–C3	1.460(2)	1.461(2)	1.463(2)	1.467(2)	1.463(2)
N2–C4	1.361(2)	1.362(2)	1.366(2)	1.367(2)	1.370(1)
C4–C5	1.450(2)	1.456(2)	1.460(2)	1.459(2)	1.457(2)
C5–N3	1.281(2)	1.284(2)	1.277(2)	1.281(2)	1.278(1)
N3–C6	1.410(2)	1.421(2)	1.427(2)	1.419(2)	1.412(2)
C_arc_–C_ar_^b^	1.395(2)	1.400(2)	1.399(2)	1.402(2)	1.398(2)
C7–O1	1.352(1)	1.354(2)	1.369(2)	1.357(2)	1.359(2)

**Bond angles (°)**					
C4–C5–N3	122.4(1)	123.4(1)	123.2(1)	122.3(1)	118.4(1)
C5–N3–C6	122.4(1)	120.8(1)	118.2(1)	123.4(1)	122.7(1)
C1–N1–C4	106.0(1)	106.2(1)	105.6(1)	106.2(1)	105.5(1)
C2–N2–C4	106.8(1)	107.0(1)	107.1(1)	107.0(1)	107.1(1)
N3–C6–C7	126.7(1)	126.8(1)	127.3(1)	121.9(1)	127.0(1)
N3–C6–C11	115.1(1)	114.6(1)	114.4(1)	118.8(1)	114.0(1)

a The mean for molecules (1a) and (1b) is reported due to their similarity.

b Mean C–C bond lengths of the phenyl rings.

### 2.2 General experimental method for (1)–(3)

Compounds (1) and (2) were synthesised based on a generic condensation reaction in ethanol.^30^ A solution of 1-methyl-2-imidazolecarboxaldehyde (3.00 mmol) in ethanol (10 mL) was added to a solution of the corresponding aminophenol (3.00 mmol): 2-amino-4-methylphenol and 2-amino-4-*tert*-butylphenol for (1) and (2), respectively. The resulting yellow solution was heated to reflux for 90 minutes. The solvent was removed *via* rotary evaporation under reduced pressure and the resulting orange oil cooled to −20 °C for 12 hours, yielding yellow crystals. Compound (3) was synthesised *via* a solid-state reaction, which has been used to prepare related imine compounds,^31^ followed by a re-crystallisation process using toluene. 1-Methyl-2-imidazolecarboxaldehyde (4.42 mmol) and 2-aminophenol (2.99 mmol) were ground to a paste in an agate pestle and mortar for approximately 10 minutes. Upon standing, a yellow powder formed which was then dissolved in toluene (15 mL) with activated 3 Å molecular sieves. The solution was heated to reflux for 30 minutes. The molecular sieves were filtered from the hot solution. The yellow filtrate was left to stand at room temperature overnight. Dark yellow hexagonal-shaped crystals (0.352 g) formed upon slow evaporation of the solvent. The crystals were collected by filtration, crushed and placed in a vacuum oven at 50 °C for 2 hours. A second re-crystallisation gave a final yield of 71%.

#### 2.2.1 4-Methyl-2-{[(*E*)-(1-methyl-1*H*-imidazol-2-yl)methylidene]amino}phenol (1)

**Fig. 1:**
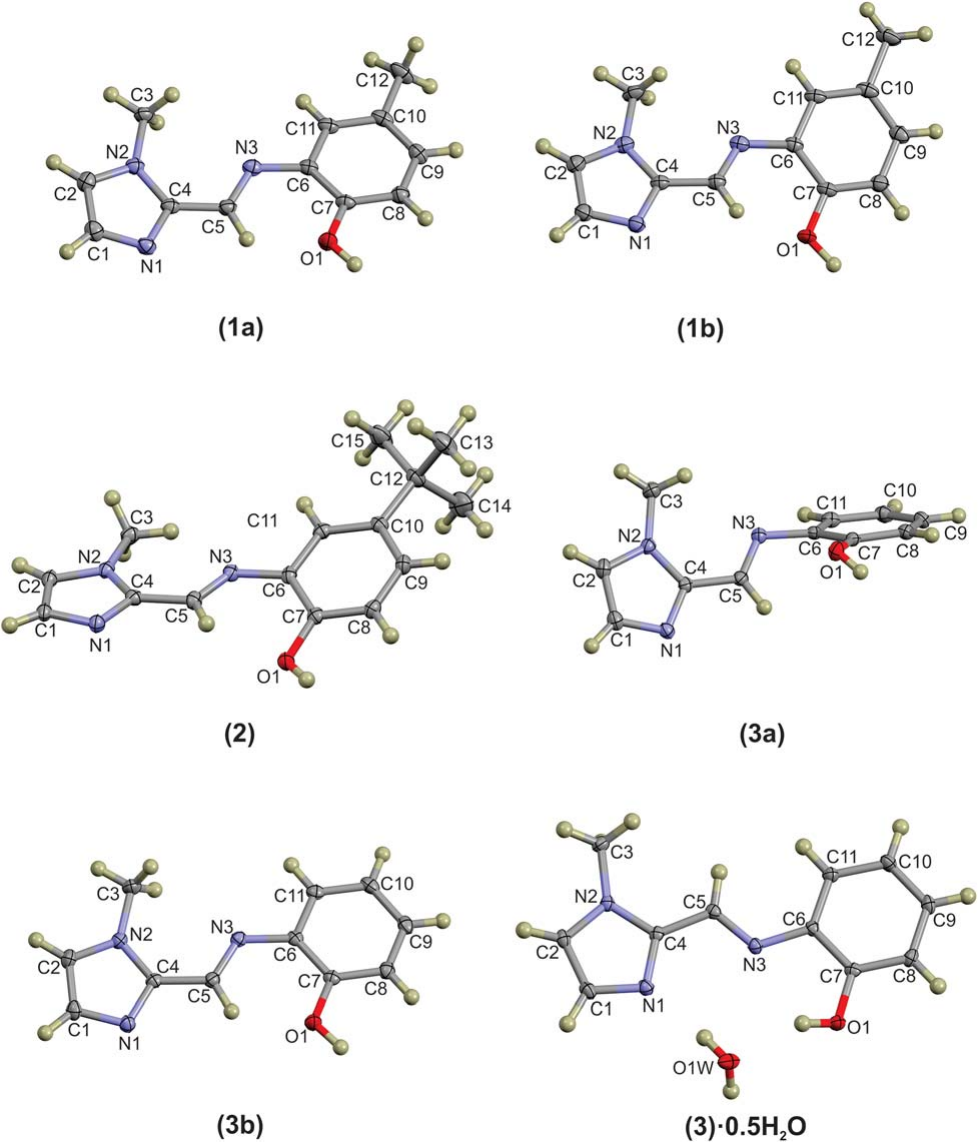
Thermal displacement plots (50% probability) showing the atom numbering schemes of compounds (1)–(3) and (3)·0.5H_2_O. Hydrogen atoms are shown as spheres of arbitrary radius. The symmetry-completed (two-fold rotational symmetry) water molecule of (3)·0.5H_2_O is shown.

Cooling the oil of (1) yielded yellow crystals suitable for X-ray diffraction. Yield: 0.605 g, 94%. UV-vis: (CH_3_CN) *λ*_max_ [nm] (*ε*/M^−1^ cm^−1^): 298 (1.60 × 10^4^), 357 (1.65 × 10^4^). Anal. calc. for C_12_H_13_N_3_O: C, 66.95; H, 6.09; N, 19.53%. Found: C, 66.69; H, 5.77; N, 19.16%. ESI-MS in methanol: [M + Na]^+^ 238.0959 *m*/*z*. Refer to Fig. 1 for the numbering scheme used in the NMR assignments. ^1^H NMR (400 MHz, DMSO-*d*_6_, 303 K) [*δ*, ppm]: 8.76 (s, br, 1H, *OH*), 8.54 (s, 1H, N=C–*H*), 7.40 (s, br, 1H, *H*-2), 7.14 (s, br, 1H, *H*-1), 6.95 (d, 1H, *H*-7), 6.88 (dd, 1H, *H*-9), 6.79 (d, 1H, *H*-10), 4.07 (s, 3H, N–*CH*_3_), 2.23 (s, 3H, C–*CH*_3_). ^13^C NMR (100 MHz, DMSO-*d*_6_, 303 K) [*δ*, ppm]: 150.70 (*C*_5_), 148.21(*C*_11_), 143.04 (*C*_3_), 137.67 (*C*_6_), 129.42 (*C*_1_), 128.25 (*C*_8_), 127.66 (*C*_9_), 126.28 (*C*_2_), 120.25 (*C*_7_), 115.96 (*C*_10_), 35.23 (*C*_4_), 20.07 (*C*_12_). IR (cm^−1^): 3137 (O–H), 1594 (C=N), 1424, 1406 (C=C phenol), 754 (imidazole C–H bend). Mp: 129.3–129.9 °C.

#### 2.2.2 4-*tert*-Butyl-2-{[(*E*)-(1-methyl-1*H*-imidazol-2-yl)methyl idene]amino}phenol (2)

Slow evaporation of (2) from a THF/hexane solution yielded yellow crystals suitable for X-ray diffraction. Yield: 0.663 g, 86%. UV-vis: (CH_3_CN) *λ*_max_ [nm] (*ε*/M^−1^ cm^−1^): 296 (1.28 × 10^4^), 355 (1.23 × 10^4^). Anal. calc. for C_15_H_19_N_3_O: C, 70.01; H, 7.44; N, 16.33%. Found: C, 70.10; H, 7.17; N, 16.13%. ESI-MS in methanol: [M − H]^−^ 256.1446 *m*/*z*. ^1^H NMR (400 MHz, DMSO-*d*_6_, 303 K) [*δ*, ppm]: 8.78 (s, br, 1H, *OH*), 8.56 (s, 1H, N=C–*H*), 7.41 (s, br, 1H, *H*-2), 7.14 (s, br, 1H, *H*-1), 7.09 (m, 2H, *H*-7 and *H*-9), 6.82 (m, 1H, *H*-10), 4.08 (s, 3H, N–*CH*_3_), 1.27 (s, 9H, C(*CH*_3_)_3_). ^13^C NMR (100 MHz, DMSO-*d*_6_, 303 K) [*δ*, ppm]: 150.94 (*C*_5_), 147.99 (*C*_11_), 143.07 (*C*_3_), 141.96 (*C*_8_), 137.38 (*C*_6_), 129.38 (*C*_1_), 126.24 (*C*_2_), 123.86 (*C*_7_), 116.87 (*C*_9_), 115.61 (*C*_10_), 35.26 (*C*_4_), 33.81 (*C*_12_), 31.31 (*C*_13_). IR (cm^−1^): 3140 (O–H), 1618 (C=N), 1436 (C=C phenol), 759 (imidazole C–H bend). Mp: 136.2–136.4 °C.

#### 2.2.3 2-{[(*E*)-(1-Methyl-1*H*-imidazol-2-yl)methylidene]amino} phenol(3)

Slow evaporation of (3) from a toluene solution yielded dark yellow crystals. Yield: 0.425 g, 71%. UV-vis: (CH_3_CN) *λ*_max_ [nm] (*ε*/M^−1^ cm^−1^): 297 (1.07 × 10^4^), 348 (1.34 × 10^4^). Anal. calc. for C_11_H_11_N_3_O: C, 65.65; H, 5.51; N, 20.89%. Found: C, 65.63; H, 5.24; N, 20.85%. ESI-MS in DMSO: [M + Na]^+^ 224.0801 *m*/*z*. ^1^H NMR (500 MHz, DMSO-*d*_6_, 303 K) [*δ*, ppm]: 8.97 (s, br, 1H, *OH*), 8.54 (s, 1H, N=C–*H*), 7.41 (s, br, 1H, *H*-2), 7.14 (d, 1H, *H*-1), 7.13 (dd, 1H, *H*-7), 7.08 (m, 1H, *H*-9), 6.91 (dd, 1H, *H*-10), 6.84 (m, 1H, *H*-8), 4.08 (s, 3H, N–*CH*_3_). ^13^C NMR (125 MHz, DMSO-*d*_6_, 303 K) [*δ*, ppm]: 150.93 (*C*_5_), 150.53 (*C*_11_), 143.02 (*C*_3_), 138.14 (*C*_6_), 129.45 (*C*_1_), 127.26 (*C*_9_), 126.33 (*C*_2_), 119.79 (*C*_7_), 119.61 (*C*_8_), 116.14 (*C*_10_), 35.24 (*C*_4_). IR (cm^−1^): 3140 (O–H), 1634 (C=N), 1450, 1431 (C=C phenol), 755 (imidazole C–H bend). Mp: 120.1–120.7 °C.

## 3. Results and discussion

### 3.1 Synthesis of ligands (1)–(3)

Three *O*,*N*,*N*′ tridentate imidazole–imine Schiff base ligands have been designed and synthesised for chelation to the VO^2+^ metal centre. The ligands comprise a 1-methyl-1*H*-imidazole ring linked by an imine bond at the 2-position to either 2-amino-4-methylphenol (1), 2-amino-4-*tert*-butylphenol (2), or 2-aminophenol (3). Compounds (1) and (2) are novel. Compound (3) is known,^20^ however no characterisation data or crystal structure of the free ligand has been reported. All ligands were synthesised by a condensation reaction of 1-methyl-2-imidazolecarboxaldehyde with the corresponding aminophenol. A common synthetic route for this condensation reaction is to reflux the carboxaldehyde and amine in ethanol or methanol and allow the imine ligand to precipitate upon cooling.^30,33^ This method did not produce a precipitate from the mother liquor and so all solvent was removed; this yielded the target compound as an oil. The formation of an oil is not unusual for this class of compound.^10,14,16,18,19^ Upon cooling to −20 °C, crystals of the respective ligand formed which remained stable once warmed to room temperature. The product yields were high (94% and 86% for (1) and (2), respectively) and of excellent purity (see ESI†^1^H and ^13^C NMR spectra). Attempts to synthesise (3) utilising this same procedure did not yield the target compound. Synthesis of (3) has been reported.^20,25^ However, an efficient solid-state synthesis adapted from Akerman and Chiazzari^31^ was employed to synthesise (3) due to the reduced number of synthetic steps and minimal solvent use, *i.e.* a green synthetic method. In the adapted method, the 2-aminophenol and a slight excess of 1-methyl-2-imidazolecarboxaldehyde were ground to a paste in an agate pestle and mortar with no additional solvents. The formation of the paste is indicative of the elimination of water, concomitant with imine bond formation. The target compound was most effectively recrystallized from toluene. The imidazole starting material, which is soluble in toluene, was added in excess to ensure complete reaction of the 2-aminophenol for which the solubility in toluene is similar to that of the target compound. Using the solubility differences was a simple and effective purification technique. When the re-crystallisation process was performed at room temperature, the crystals formed were the hemihydrate ((3)·0.5H_2_O), even with the use of molecular sieves in the re-crystallisation process. The anhydrous compound (3) could be obtained by dissolving the yellow powder formed from the solid-state reaction in toluene with activated 3 Å molecular sieves and heating the resulting solution for 30 minutes. Crystals of (3) formed upon slow cooling the hot solution.

### 3.2 NMR spectroscopy

The imine group has similar chemical shifts in all three compounds in both the ^1^H (8.54 ppm for (1) and (3) and 8.56 ppm for (2)) and ^13^C NMR spectra (150.70, 150.94 and 150.93 ppm for (1), (2) and (3), respectively). The same is true for the imidazole ^1^H and ^13^C chemical shifts. The ^1^H NMR chemical shifts for the imine and hydroxyl group showed that, as in the solid state, the OH hydrogen atom remained on the oxygen in solution and did not migrate to the imine nitrogen to switch from the enol to the keto tautomer as has been reported for similar compounds.^30^ The chemical shift for the OH group is similar for compounds (1) and (2) (8.76 ppm and 8.78 ppm, respectively), but is further downfield for compound (3) at 8.98 ppm. The electron-donating effect of the methyl and *tert*-butyl moieties *para* to the OH group have a shielding effect while the un-substituted phenol ring which has the electron withdrawing effect of the phenyl ring is deshielded in comparison. A similar effect is noted in the ^13^C chemical shifts of the *C*–OH atom (148.21, 147.99 and 150.53 ppm for compounds (1), (2) and (3), respectively). Fully assigned ^1^H and ^13^C spectra are available in the ESI.†

### 3.3 X-ray crystallography of compounds (1)–(3) and (3)·0.5H_2_O

Compounds (1)–(3) and (3)·0.5H_2_O were all studied by single crystal X-ray diffraction. Crystal data and structure refinement details are summarised in Table 1. The thermal displacement plots of (1), (2), (3) and (3)·0.5H_2_O are shown in Fig. 1.

Compound (1) crystallises in the monoclinic space group *P*2_1_/*n* with two hydrogen-bonded molecules comprising the asymmetric unit. The complementary hydrogen bonding between the imidazole N atom and the OH group of the adjacent molecule leads to a 16-membered hydrogen-bonded ring. The hydrogen bond parameters are summarised in Table 3. Despite the extended aromaticity of the ligands, the molecules exhibit a notable deviation from planarity. This deviation from planarity is indicated with the angle subtended by the five- and six-atom mean planes of the imidazole and phenyl rings, respectively. This measures *ca.* 15° for both molecules (1a) and (1b) of the asymmetric unit. The most significant difference in geometry between the two molecules in the asymmetric unit of (1) is the direction of the relative rotation between the phenyl and imidazole rings. With the phenyl ring as a reference, the methylimidazole moiety is rotated below the mean plane of the phenyl ring in one case and above in the second. The bond lengths and angles describing the imine bond for each of the molecules are summarised in Table 2. Compound (2) crystallised in the *C*2/*c* space group with a single molecule in the asymmetric unit. Despite the significant difference in steric bulk between the methyl and *tert*-butyl substituents of compounds (1) and (2), the geometry of the molecules is remarkably similar. The same distortion from planarity is noted in compound (2) with the angle subtended by the imidazole and phenyl rings measuring *ca.* 22°. The energetics of these out-of-plane rotations is further explored using molecular simulations (*vide infra*).

**Table 3:** Hydrogen bond lengths (Å) and bond angles (°) describing the stabilising intermolecular interactions for (1)–(3) and (3)·0.5H_2_O^a^

Bond	D–H (Å)	H⋯A (Å)	D⋯A (Å)	D–H⋯A (°)
(1)				
O1A–H101⋯N1B	0.93(2)	1.76(2)	2.672(2)	169(2)
O1B–H102⋯N1A	0.99(2)	1.69(2)	2.662(2)	166(2)

(2)				
O1–H101⋯N1	0.98(3)	1.70(3)	2.667(2)	168(3)

(3)				
O1A–H101⋯N1A^i^	0.89(2)	1.81(2)	2.688(1)	170(2)
O1B–H102⋯N1B^ii^	0.95(2)	1.75(2)	2.672(1)	164(1)

(3)·0.5H_2_O				
O1–H101⋯O1S	0.83(2)	2.18(2)	2.936(1)	151(2)
O1S–H1S⋯N1	0.89(2)	1.95(2)	2.832(1)	173(2)

a Symmetry operators: (i) 1 − *x*, 2 − *y*, −*z*; (ii) −*x*, 1 − *y*, −*z*.

Compound (3) has been studied in the solid state as both the hydrated and anhydrous forms: (3)·0.5H_2_O and (3), respectively. Some significant variations in the geometry are evident. In compound (3), which crystallised in the *P*2_1_/*c* space group, the asymmetric unit comprises two independent molecules with significantly different molecular configurations. Molecule (3a) shows a more perpendicular orientation of the phenyl and imidazole rings while (3b) is approximately planar. Using the same six- and five-membered phenyl and imidazole mean planes to describe the deviation from planarity, the angles subtended by molecules (3a) and (3b) measure approximately 66° and 7°, respectively. The hydrated form crystallised in the monoclinic *C*2/*c* space group and has adopted a configuration with a slight distortion from planarity (as indicated by an angle of 6.1° between the phenyl and imidazole mean planes), consistent with compounds (1), (2) and (3b).

Selected bond lengths for the compounds are summarised in Table 2. The C5–N3 imine bond lengths, which range from 1.277–1.284 Å and the C3–C5–N3 bond angles, which range from 122.3–123.4° are indicative of the double bond character of the azomethine group and sp^2^ hybridisation of the imine carbon atom. The isomerisation about the imine bond is exclusively *trans* for all compounds studied. The *trans* configuration is seemingly favoured as there would be non-bonded repulsion between the hydroxyl and imidazole N–CH_3_ group in a *cis* configuration. The *trans* configuration also allows for weakly stabilising intramolecular C–H⋯O interactions. This *trans* configuration has been reported for similar compounds.^10–20,32^ The bond distances and angles show little variation within the present library of compounds and compare favourably with those previously reported for related compounds.^10–20^ The data in Table 2 show that the N3–C6–C7 and N3–C6–C11 bond angles are (unexpectedly) significantly different, measuring *ca*. 127 and 114°, respectively. This deviation from the ideal angle of 120° for an sp^2^ hybridised carbon atom is likely a consequence of steric repulsion between the phenol OH group and the imine C–H. This distortion is less pronounced for molecule A of compound (3) as the steric strain is released by the out-of-plane rotation of the phenyl ring.

**Fig. 2:**
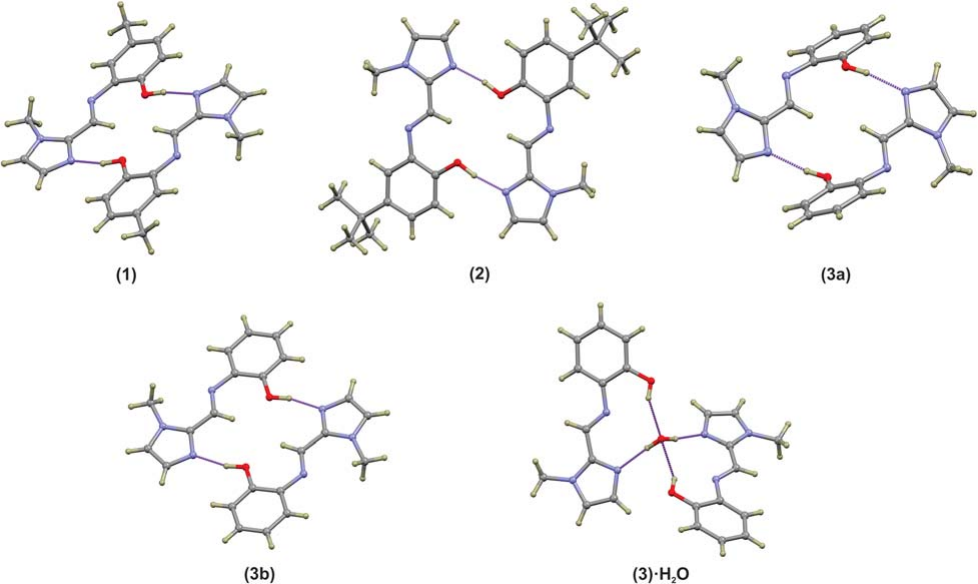
Dimeric structures of (1), (2), (3a) and (3b) as well as the water-bridged dimer of (3)·0.5H_2_O. In compounds (1)–(3), the same general complementary hydrogen bonding leads to a 16-membered hydrogen-bonded ring. The bridged hydrate has two 10-membered hydrogen-bonded ring structures. The hydrogen bonds are shown as dashed purple lines. Atoms are shown as spheres of arbitrary radius.

Compounds (1)–(3) form complementary hydrogen bonds between the un-substituted imidazole nitrogen and the OH group of the neighbouring molecule. This hydrogen bonding motif yields a dimeric supramolecular structure supported by a sixteen-membered hydrogen bonding ring. In the case of (1) the asymmetric unit consists two molecules which are linked by hydrogen bonds while ligand (2) has a single molecule in the asymmetric unit. The asymmetric unit of the anhydrous compound (3) comprises two symmetry-independent molecules which are not hydrogen-bonded to each other, but rather to neighbouring molecules. The most notable difference between the two independent molecules in the asymmetric unit in compound (3) is the C5–N3–C6–C7 torsion angle. For the relatively planar molecule A this torsion angle is −7.7(2)° and for molecule B, with the out-of-plane twisting of the phenyl ring, it measures −59.1(2)°. Despite the differences in molecular geometry, the same complementary hydrogen bonding motif is evident in both structures. The dimeric supramolecular structures are shown in Fig. 2. The hydrogen bonding parameters are summarised in Table 3.

**Fig. 3:**
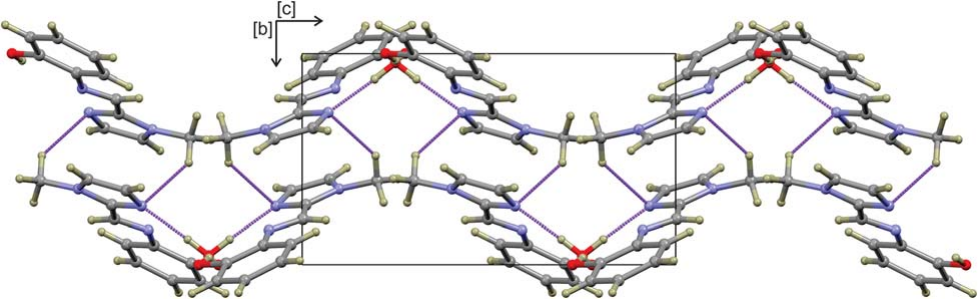
One-dimensional supramolecular structure of (3)·0.5H_2_O viewed down the *a*-axis. The structure is co-linear with the *c*-axis and comprises water-bridged hydrogen-bonded dimers cross-linked by C–H⋯N interactions. All atoms are shows as spheres of arbitrary radius and intermolecular interactions are shown as dashed purple lines.

The hydrogen-bonded dimers in (1) are symmetry independent, but in (2) and (3) inversion dimers are formed. The water-bridged dimer of (3)·0.5H_2_O is of *C*_2_ symmetry. These hydrogen bonds are considerably shorter than the sum of the van der Waals radii of the interacting atoms. This short bond length coupled with the fact that the bonds are approaching the ideal bond angle would suggest that they are moderate to strong interactions. The compounds also all show intramolecular interactions between the imine C–H (donor) and the phenol OH (acceptor) group. The dimers of (3a) and (3b) are linked by C–H⋯O interactions between the phenol OH group and the imidazole C–H group to form one-dimensional columns which transverse the *ab* plane. The one-dimensional column is shown in Fig. S7.† In the case of (3)·0.5H_2_O, the water-bridged dimers are linked through C–H⋯N interactions to form a one dimensional column co-linear with the *c*-axis (Fig. 3). The same imidazole N atom therefore acts as an acceptor for two intermolecular interactions. For compounds (1), (2) and (3) the imine N atom is precluded from participating in intermolecular interactions because of steric crowding by the imidazole methyl group and *ortho* hydrogen atom of the phenol ring.

### 3.4 Solution studies

**Fig. 4:**
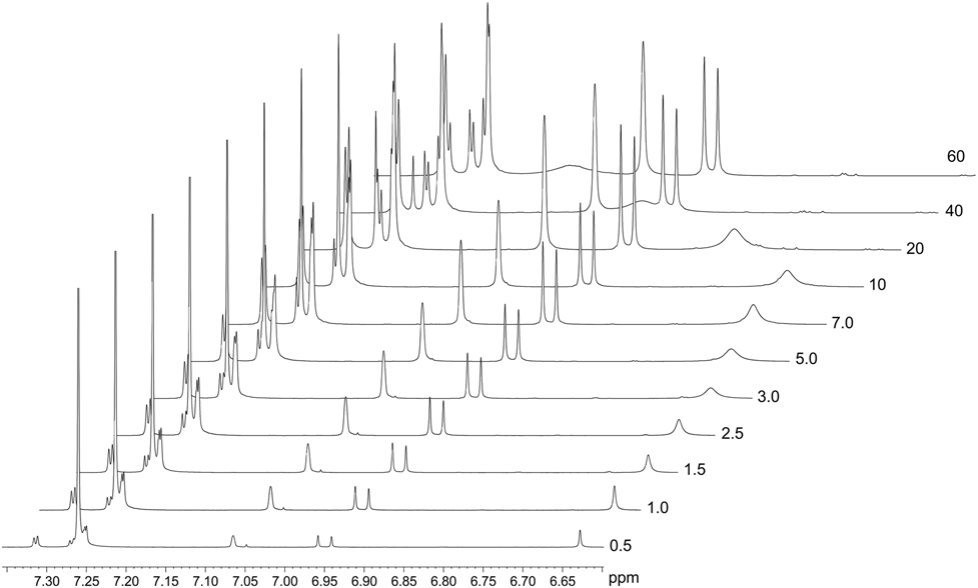
Stacked ^1^H NMR spectra of (2) depicting the concentration dependence of both the chemical shift and line width of the OH signal: original chemical shift 6.62 ppm (3.24 × 10^−3^ M) and final chemical shift of 7.11 ppm (3.88 × 10^−1^ M). These spectral changes are a consequence of hydrogen bonding (dimerization) in solution.

The spontaneous dimerization of compounds (1)–(3) in the solid state was established through X-ray crystallography. This then begs the question whether the same process is apparent in the solution phase. To this end, the dimerization process at 30 °C was followed by ^1^H NMR titration. Compound (2) was selected for the solution phase study as it shows the highest solubility in chloroform owing to the *tert*-butyl functional group. ^1^H NMR spectra for compound (2) were recorded at 30 °C over a concentration range of *ca.* 3 × 10^−3^–4 × 10^−1^ M based on the mass of the monomeric unit. The line shapes and chemical shifts of the OH group were followed over this concentration range (Fig. 4). The chemical shift of the OH group varies significantly over this concentration range, moving downfield from 6.62 ppm to 7.11 ppm. This shift is likely a consequence of the deshielding effect of hydrogen bonding. Importantly, the chemical shifts and line widths of the remaining peaks are unchanged over this concentration range. This shows that their magnetic environment does not change with concentration. These signals are therefore a reference point for the ^1^H NMR spectrum of (2) and highlight the marked change of the phenolic OH group as a consequence of intermolecular hydrogen bonding. In addition to the deshielding of the OH group, line broadening is also noted. This is a consequence of the greater rate of exchange for the monomer ↔ dimer equilibrium, in relation to the timescale of the 500 MHz ^1^H NMR. The observed signal is the weighted time average of the two species in solution. The observed resonance may then be explained by the simple sum of the products of the line widths and fractions of the monomer and dimer as shown in eqn (1).

1 *δ*_obs_ = (*δ*_M_ × *f*_M_) + (*δ*_D_ × *f*_D_)

In eqn (1), *δ*_obs_ is the observed chemical shift, *δ*_M_ and *f*_M_ are the chemical shift and fraction of the monomeric species, respectively. Similarly, *δ*_D_ and *f*_D_ are the chemical shift and fraction of the dimeric species, respectively, in solution at a given concentration.

The concentration-dependent ^1^H NMR spectra strongly suggest that the dimerization process does take place in the solution state. To quantify this, the equilibrium constant for the dimerization process was established by monitoring the change in chemical shift of the OH group as a function of concentration. By fitting eqn (2), the equilibrium constant for the dimerization process was determined.^33^Eqn (2) is a 1 : 1 binding equation, which is appropriate for a dimerization process,^33^ and was fitted by a least squares process using Origin 9.1.2
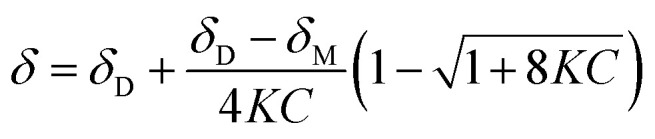


**Fig. 5:**
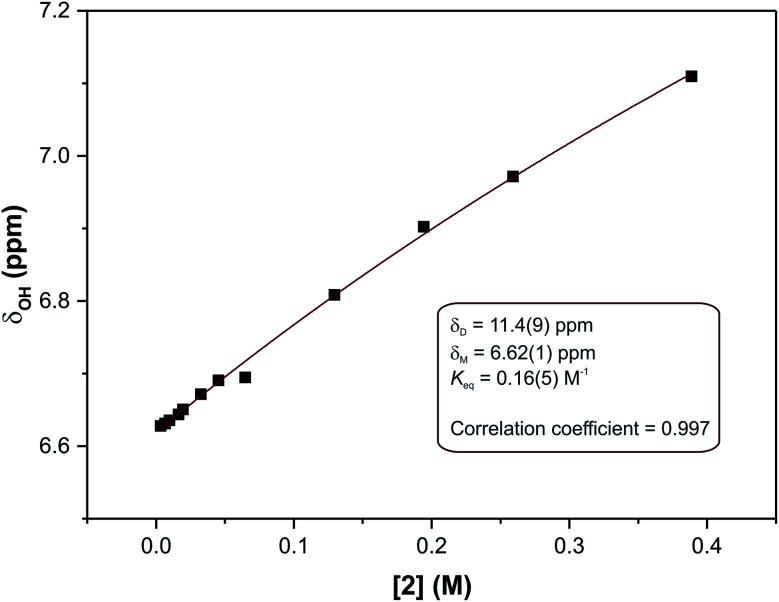
Non-linear least squares fit of eqn (2) to the concentration-dependent phenolic OH chemical shift in the ^1^H NMR spectra of compound (2). The result is an equilibrium constant of 0.16(5) M^−1^.

In eqn (2), *δ* is the weighted average chemical shift of the phenolic OH hydrogen (*i.e.* the observed chemical shift), *δ*_D_ is the chemical shift of the OH group in the dimeric species, *δ*_M_ is the chemical shift of the OH hydrogen atom in the monomeric species, *K* is the equilibrium constant and *C* is the formal concentration of the monomer in solution. The fitted data are presented in Fig. 5.

The non-linear least squares fit of eqn (2) to the concentration-dependent ^1^H NMR chemical shift of the phenolic OH group in Fig. 5 shows that the equilibrium constant for the process is 0.16(5) M^−1^ at 30 °C with *δ*_D_ and *δ*_M_ values estimated to be 11.4(9) and 6.62(1) ppm, respectively, at the same temperature. The Δ*G* value for this reaction is *ca.* 4.6 kJ mol^−1^. This shows that at this temperature the process is slightly endergonic and may explain why relatively high concentrations of compound (2) were required before a significant change in the chemical shift of the OH group was observed. These data are in agreement with the energetics of similar systems.^33^ The Δ*G* value is relatively close to zero in CDCl_3_ at 30 °C, without variable temperature studies it is not possible to know whether the dimerization process would remain endergonic at lower temperatures or become exergonic. In the solid state, the hydrogen bond parameters and molecular geometry are very similar irrespective of the functional group on the phenol ring. Speculatively, the dimerization processes for compounds (1) and (3) in the solution state should therefore have similar equilibrium constants to compound (2).

To ensure that the observed spectral changes were due to the dimerization process and not hydrogen bonding to water, the effects of water on the spectra were also examined through the addition of small aliquots (1 μL) of water to the NMR samples. This did lead to some line broadening, but importantly had minimal effect on the chemical shift which moved downfield ≪1%. This suggests, firstly, that the process of drying the CDCl_3_ over CaH_2_ was effective and that, secondly, the changes in chemical shift as a function of concentration are the result of Schiff base dimerization in solution.

### 3.5 Density functional theory

The solid-state structures showed some unexpected molecular configurations for the different compounds. This prompted a further study into the optimum geometry and relative energies of these molecular configurations. Molecular simulations using Density Functional Theory (DFT) were performed using Gaussian 09 W.^34^ The X-ray coordinates were used for the input structures unless otherwise specified. Lowest energy conformations were determined through geometry optimisation of both the dimeric and monomeric structures. Frequency simulations were completed for both the monomeric and dimeric species of each compound. All simulations were run at the B3LYP/6-311G level of theory, single first polarisation and diffuse functions (d,p) were also added to the basis set to improve accuracy. Input files were prepared, and output files analysed using GaussView 5.0.^35^ The frequency simulations suggest the geometry optimisations are true minima on the global potential energy surface based on a lack of negative frequency eigenvalues. Transition energies and oscillator strengths were calculated for 24 excited states using the TD-DFT method^36–44^ at the same level of theory applied to the geometry optimisations. A Polarizable Continuum Model (PCM) was included in the calculation of transition energies and oscillator strengths to account for any solvent effects.^43,44^ The TD-DFT simulations were performed on the *in vacuo* geometry-optimised structures. The molecular orbitals were assigned by studying the spatial distribution of their isosurfaces. Only singlet excited states were considered. The experimental absorption spectra were recorded in acetonitrile and thus an acetonitrile solvent continuum was included in the simulations to account for any possible solvent effects.

**Fig. 6:**
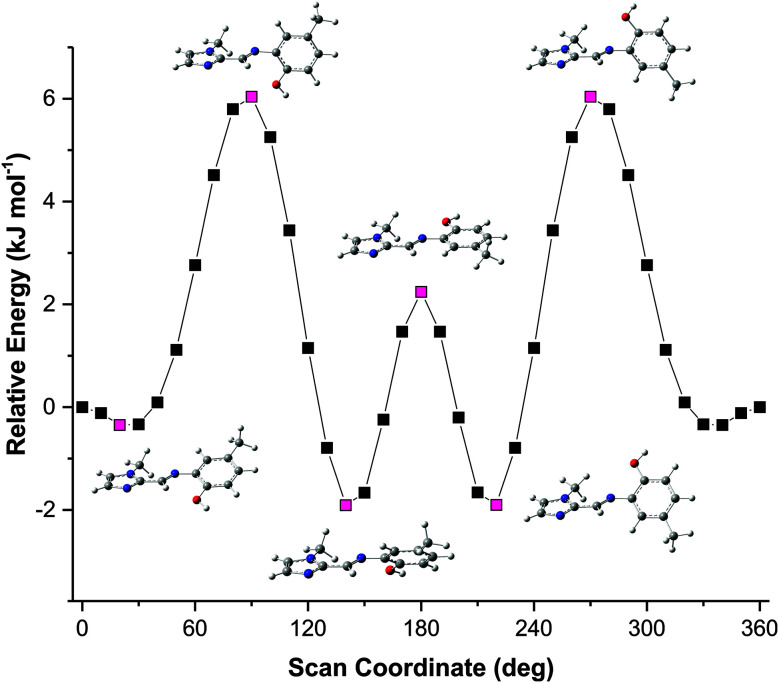
Relative energy of compound (1) as a function of the C5–N3–C6–C7 torsion angle. The energy maxima and minima are highlighted in magenta with their corresponding structures. The simulations were run using a scan operation with 36 steps at 10° intervals. The conformation with a torsion angle of 0° is used as the energy reference point.

The solid-state structure of compound (3) showed that two distinct molecular configurations exist in the solid state. This prompted a study of the relative energies of these configurations. In addition to the relative energies of these two configurations, the total energy of the molecules as a function of the relative angles of the phenyl and imidazole rings was performed (rotations were effected in 10° increments around the C5–N3–C6–C7 torsion angle). These data showed an interesting relationship between energy and molecular geometry. This relationship is illustrated in Fig. 6. The same relationship between molecular geometry and total energy was noted for compounds (1)–(3), irrespective of the substitution on the phenyl ring. This result seems reasonable as the relative rotation of the phenyl ring with substitution at the 4-position will not be influenced by steric interactions *in vacuo*.

**Fig. 7:**
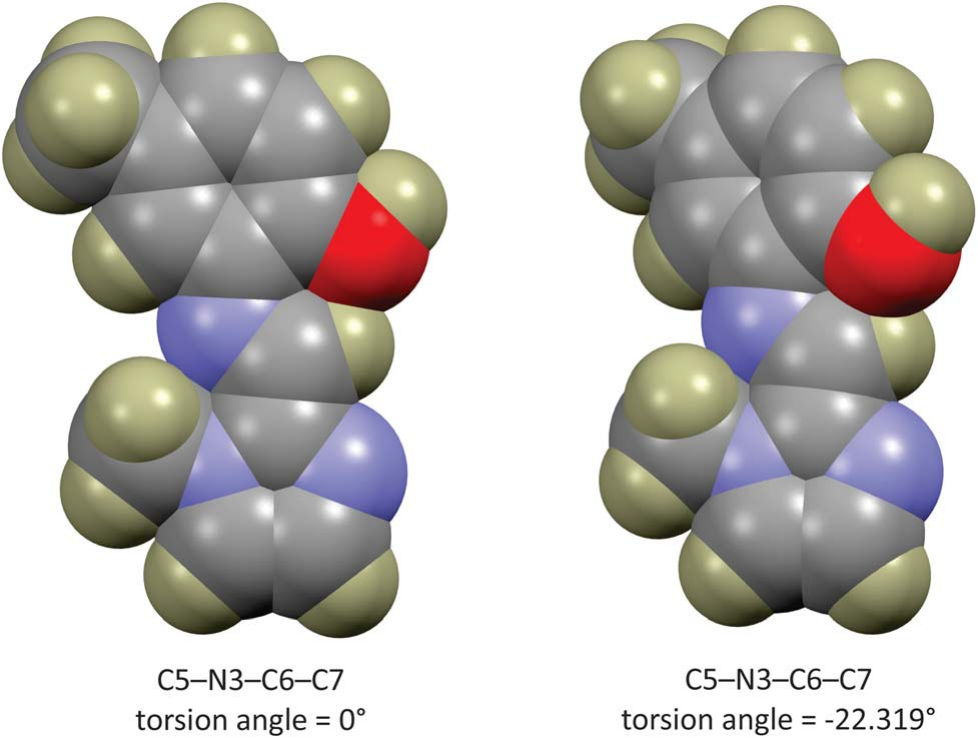
‘Space filling’ plot for compound (1) showing all atoms rendered at their van der Waals radii. The plot shows that the out-of-plane rotation reduces the non-bonded repulsion between the C–H and phenol oxygen atom.

The energy scan shows that the highest energy configuration has the phenyl ring perpendicular to the molecule. In this orientation the p-orbitals may no longer overlap, the extended aromaticity of the ligands is thus broken, and the molecule is consequently destabilised. The lowest energy conformation has a C5–N3–C6–C7 torsion angle which measures 22.319°. Logically, a planar molecule which would allow for complete overlap of the p-orbitals of the conjugated π-system would be the lowest energy conformation. In practice, a slight out-of-plane rotation (as indicated by the above torsion angle) lowers the energy of the structure. A ‘space-filling’ plot which renders the atoms using their van der Waals radii (Fig. 7) provides insight into the potential reason for this. A planar configuration leads to increased steric repulsion between the imine C–H and phenol oxygen atom. An out of plane rotation increases the H⋯O distance from 2.123 to 2.205 Å, reducing the steric strain and yielding a more stable molecule. The same torsion angle measures 24.265 and 25.319° for molecules (2) and (3), respectively. The barrier to rotation of the phenol group is low, averaging 7.97 kJ mol^−1^ for the three compounds with a standard deviation of 0.06 kJ mol^−1^. This low energy barrier may indicate why compound (3) was able to adopt two quite different geometries in the solid state.

**Fig. 8:**
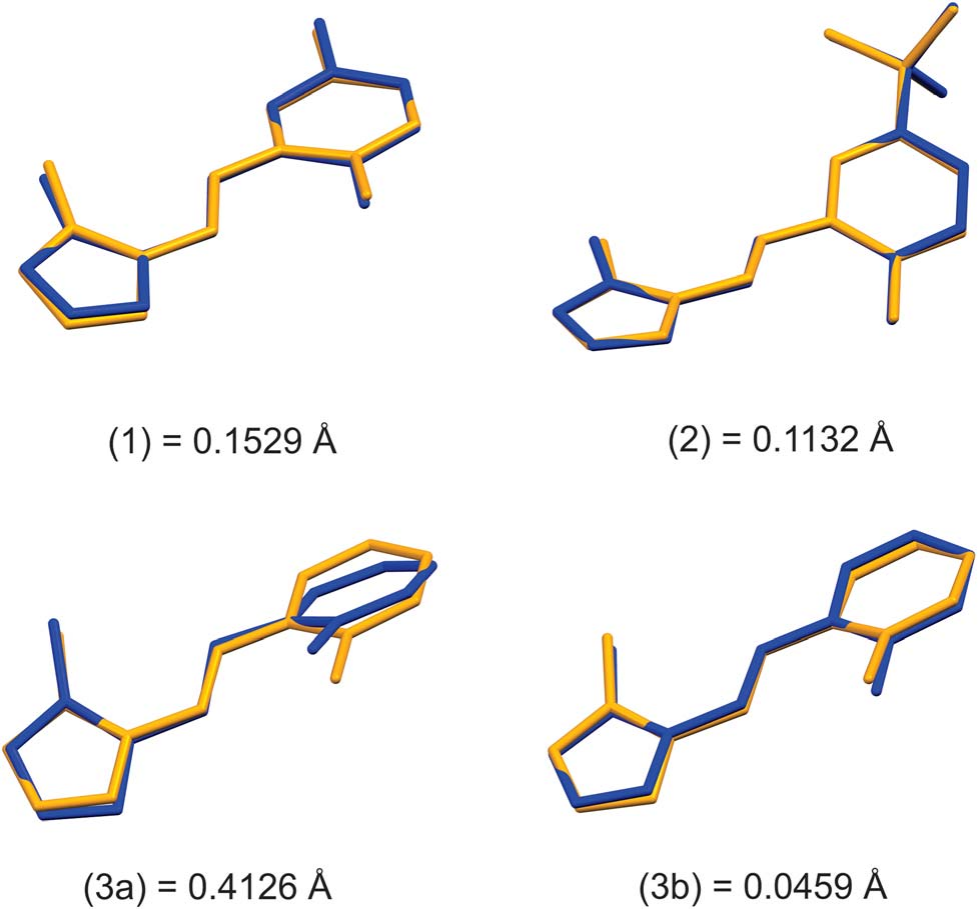
Comparison of DFT-calculated (yellow) and X-ray crystal structures (blue) of the monomeric structures of (1)–(3). Root mean square deviations (RMSD) for all non-hydrogen atoms are indicated on the diagram (Å).

**Fig. 9:**
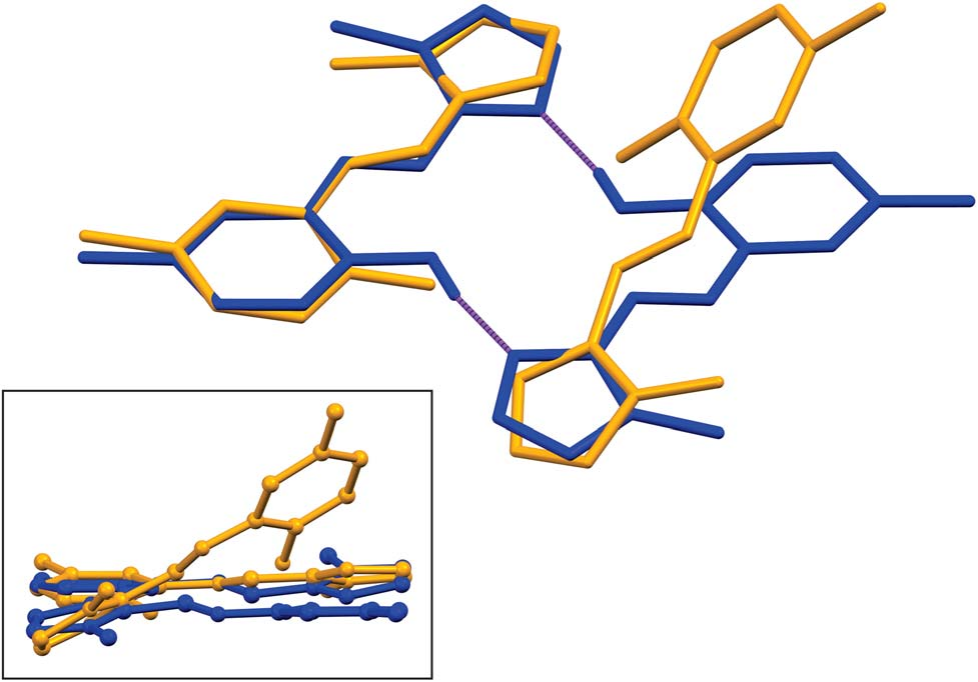
[Main] Least squares fit of a single molecule of the dimer of (1) showing the different geometries of the optimised and solid-state structures. [Inset] “Side-view” of the molecules highlighting the relative rotation of the molecules comprising the dimer. DFT-simulated molecules are shown in yellow and solid-state structures in blue.

Structural overlays (least squares fits) of geometry-optimised and experimental structures (Fig. 8) were calculated using Mercury 4.1.3.^45^ The root-mean-square deviations indicate the experimental and simulated structures are generally in good agreement for the monomers. The dimeric structures have larger deviations between the experimental and lowest energy conformations. This difference lies predominantly in the relative angle of rotation between the two molecules comprising the dimer. In compounds (1), (2) and (3b), the solid-state dimers could be considered approximately co-planar with the angles subtended by the two sixteen-atom mean plans of the non-H atoms measuring 2.0° for compound (1) and 0° for compounds (2) and (3), since they are inversion dimers. The relative rotation of the two molecules lowers the energy of the dimer by a modest 1.1 kJ mol^−1^ compared to a co-planar arrangement in the gas phase. The geometry-optimised structures show that in the absence of packing constraints imposed by a crystal lattice, the lowest energy configuration (albeit by a small margin) has the two molecules subtending angles of 39.39, 38.63 and 34.10° between the same 15-atom mean planes comprising all non-hydrogen atoms of the imidazole, phenol and imine groups. The differing geometries of the dimers are shown in Fig. 9. The hydrogen-bonded dimers formed by (2) and (3) are related by inversion symmetry while that of compound (1) is of *C*_1_ symmetry in the solid state. The geometry optimised *in vacuo* structures of all three compounds are not related through inversion symmetry, but rather *C*_2_ symmetry.

The stability gained by formation of the supramolecular structures was also calculated using the same level of theory. The hydrogen bonding motif comprises two complementary O–H⋯N hydrogen bonds. The hydrogen-bonded dimers are significantly lower in energy than the non-interacting molecules, highlighting the stability of this motif. The hydrogen bonded compounds (1)–(3) are lower in energy by 101.8(2) kJ mol^−1^. This corresponds to a bond energy of 50.9 kJ mol^−1^ per hydrogen bond of the motif. The small standard deviation shows that in the gas phase the substituent on the phenol ring has effectively no influence on the bond strength. The water-bridged supramolecular structure of (3)·0.5H_2_O is similarly stabilised by the hydrogen bonds which lower the energy by 135.1 kJ mol^−1^ compared to a model with two independent ligands and a water molecule.

In order to better understand the origins of the hydrogen bonds, the partial charge distribution (NBO charges, measured in electrons) were analysed. These indicate that the OH hydrogen atom has the highest positive partial charge in both the dimeric and monomeric species in all three compounds. The most negative partial charge is carried by the phenol oxygen atom in all species; the imidazole N atom has the second highest partial negative charge. There are examples in literature which exhibit hydrogen bonding solely between the electron-rich oxygen atom (H-bond acceptor) and the OH hydrogen atom of a neighbouring molecule (CSD reference JUBKOG).^25^ In the present structure, the added stability afforded by the complementary hydrogen bonding motif (*i.e.* two hydrogen bonds) is apparently more significant than an interaction forming merely between the species with the highest partial negative and positive charges.

**Fig. 10:**
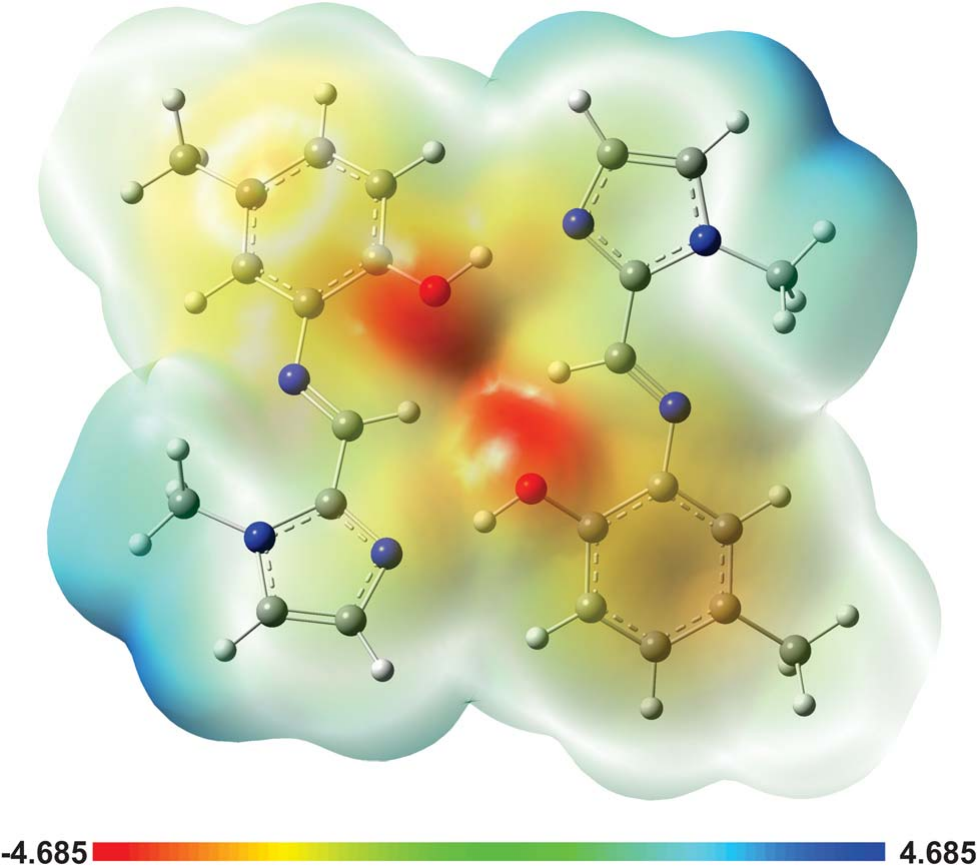
Electrostatic potential (ESP) map from the total SCF density for the dimer of (1) highlighting the zones of positive and negative potential and illustrating the origin of the hydrogen bonding.

A plot of the electrostatic potential (ESP) in Fig. 10 highlights the favourable electrostatic interaction between the imidazole N-atom and the phenolic OH group. The ESP surface also indicates formation of the intramolecular C–H⋯O interaction between the imine C–H and phenolic OH group.

The UV-visible spectra were calculated for both the monomeric and dimeric species using the TD-DFT method at the same level of theory as used for the geometry optimisations, but including an acetonitrile solvent continuum running the Polarizable Continuum Model (PCM). Table S1† indicates the main transitions with oscillator strengths for both the monomeric and dimeric structures of (1).

**Fig. 11:**
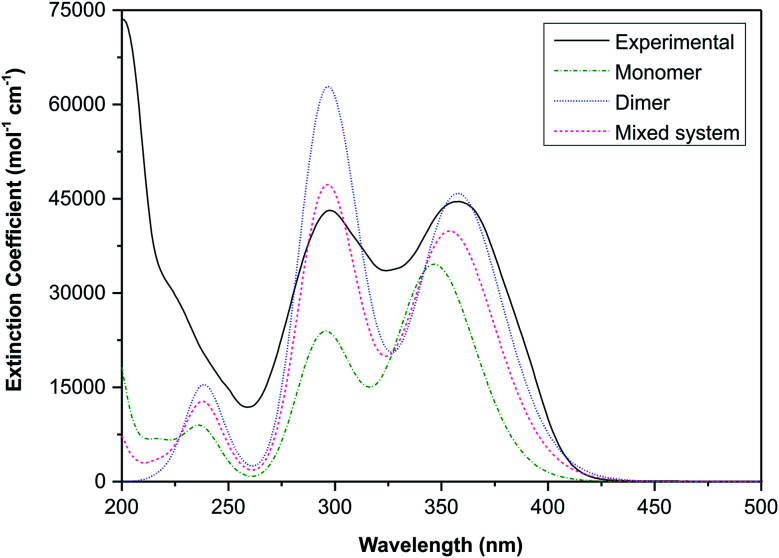
Superposition of the experimental UV-visible spectrum of (1) and the TD-DFT calculated spectra (CH_3_CN solvent continuum) of the monomeric and dimeric structures. A calculated spectrum of an equilibrium system with 60% contribution from the dimer and 40% from the monomer is also shown.

The electronic transitions are summarised in Table S1,† these data show that the spectra of both the monomeric and dimeric species are dominated by high energy π → π* transitions. To link the experimental and simulated data, a superposition plot of the spectra was generated (Fig. 11). The superposition plot shows good correlation between the experimental spectra and simulated data for the dimeric structure, particularly in terms of *λ*_max_ values. The extinction coefficients (the simulated data were not normalised) differ to some degree and suggest that the solution state is in reality an equilibrium between the monomeric and dimeric species. A calculated spectrum for a mixed system is indicated in Fig. 11, this shows excellent agreement with the experimental data for a system with 60% dimer and 40% monomer present. This result seems reasonable considering that the hydrogen-bonded supramolecular structure is more stable than the isolated molecules.

**Fig. 12:**
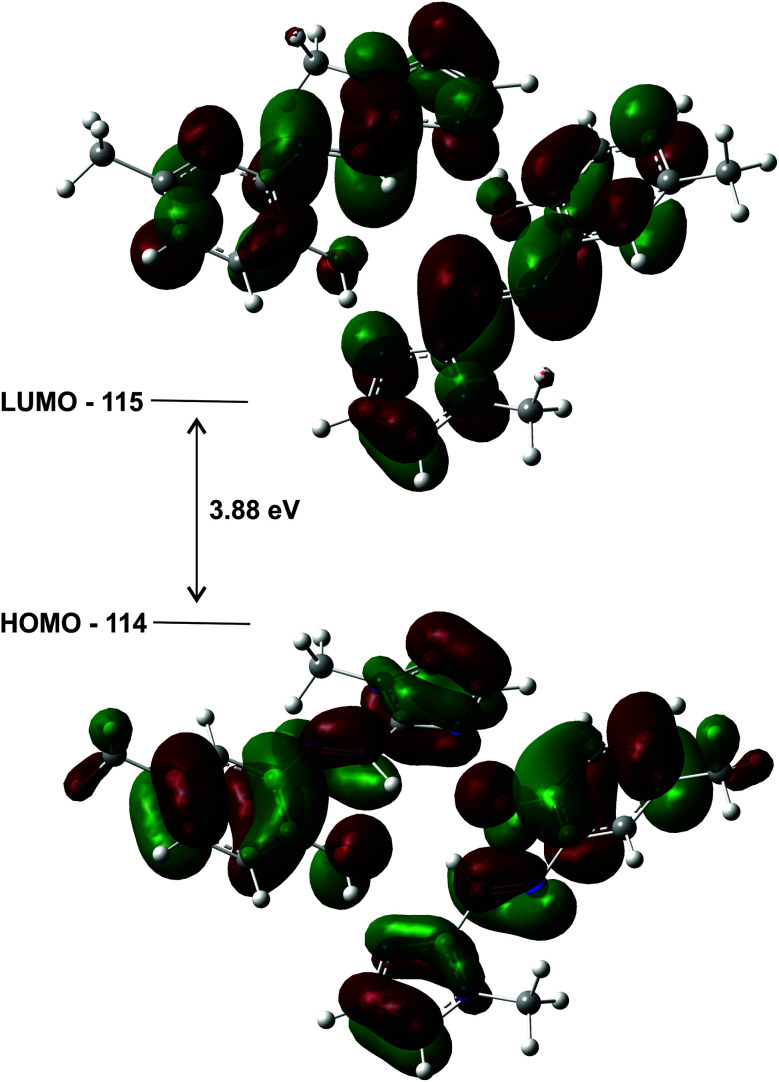
HOMO and LUMO plots for the geometry optimised (gas phase) dimer of (1) illustrating how the orbitals span both molecules. The energy gap of the frontier molecular orbitals is 3.88 eV.

The highest occupied molecular orbitals (HOMO) and lowest unoccupied molecular orbitals (LUMO) for the dimers of (1)–(3) show they are all of π-symmetry (Fig. 12), but significantly they span both molecules. This is interesting as it suggests the dimer is not simply two adjacent monomers, but rather a genuine supramolecular structure with π-electrons spread over both molecules, in accord with previous reports on H-bonded dimers of pyrrole–imine Schiff base derivatives.^31,46^ Since the basis set has been augmented with diffuse functions, the results are likely to be reliable.

## 4. Conclusions

Three *O*,*N*,*N*′ donor tridentate imidazole–imine Schiff base ligands have been synthesised and fully characterised. Ligands (1) and (2) are novel. The ligands (1) and (2) were successfully synthesised utilising a standard condensation reaction by heating to reflux 1-methyl-2-imidazolecarboxaldehyde with the corresponding phenol. Compound (3) was synthesised from 1-methyl-2-imidazolecarboxaldehyde and 2-aminophenol using a solid-state synthetic method followed by re-crystallisation from toluene. The synthetic methods proved to be rapid and high yielding and in the case of (3), environmentally friendly. The single crystal X-ray structures of (1), (2) and (3) were elucidated and showed hydrogen bonding between the un-substituted imidazole nitrogen and the OH group of a neighbouring molecule. This complementary hydrogen bonding motif leads to dimeric supramolecular structures. By varying the synthetic method, it was possible to form either the anhydrous or hemihydrate form of (3). The hemihydrate molecule favours the formation of a water-bridged supramolecular structure. The stability of the dimers was confirmed by DFT simulations which showed the hydrogen-bonded structures to be lower in energy by *ca.* 101 kJ mol^−1^ (this equates to *ca.* 50 kJ mol^−1^ for each hydrogen bond). The solid-state structures are inversion dimers for (2) and (3) and *C*_1_ symmetry for (1). The geometry optimised dimers are all of *C*_2_ symmetry. The solid-state structure of (3) showed two significantly different molecular configurations described by the relative rotation of the imidazole and phenol rings. This was probed using DFT methods which showed that the barrier to rotation for these two groups is relatively low (7.97(6) kJ mol^−1^) with the lowest energy conformations subtending angles of 22.319, 24.265 and 25.319° for molecules (1), (2) and (3), respectively. The ESP maps succinctly explain the stability of the hydrogen bonds through the partial charges of the interacting atoms. TD-DFT simulations suggest that the dimer is stable and the dominant species in solution.

## Conflicts of interest

There are no conflicts to declare.

## Supplementary Material

RA-010-C9RA10488G-s001

RA-010-C9RA10488G-s002

## References

[cit1] Demir S., Yazicilar T. K., Taş M. (2014). Inorg. Chim. Acta.

[cit2] Mandal S., Poria D. K., Seth D. K., Ray P. S., Gupta P. (2014). Polyhedron.

[cit3] Cozzi P. G. (2004). Chem. Soc. Rev..

[cit4] da Silva C. M., da Silva D. L., Modolo L. V., Alves R. B., de Resende M. A., Martins C. V. B., de Fátima Â. (2011). J. Adv. Res..

[cit5] Liu X. H., Lin L., Feng X. M. (2011). Acc. Chem. Res..

[cit6] Baleizão C., Garcia H. (2006). Chem. Rev..

[cit7] Sharma V., Piwnica-Worms D. (1999). Chem. Rev..

[cit8] Gradinaru J. A., Forni A., Druta V., Tessore F., Zecchin S., Quici S., Garbalau N. (2007). Inorg. Chem..

[cit9] Kodera M., Terasako N., Kita T., Tachi Y., Kano K., Yamazaki M., Koikawa M., Tokii T. (1997). Inorg. Chem..

[cit10] Howson S. E., Allan L. E. N., Chmel N. P., Clarkson G. J., Deeth R. J., Faulkner A. D., Simpson D. H., Scott P. (2011). Dalton Trans..

[cit11] Gu Z.-G., Pang C.-Y., Qiu D., Zhang J., Huang J.-L., Qin L.-F., Sun A.-Q., Li Z. (2013). Inorg. Chem. Commun..

[cit12] Qin L.-F., Pang C.-Y., Han W.-K., Zhang F.-L., Tian L., Gu Z.-G., Ren X., Li Z. (2015). CrystEngComm.

[cit13] Tian L., Pang C.-Y., Zhang F.-L., Qin L.-F., Gu Z.-G., Li Z. (2015). Inorg. Chem. Commun..

[cit14] Becerra A., Contreras R., Carmona D., Lahoz F. J., García-Orduna P. (2013). Dalton Trans..

[cit15] Choy S. W. S., Page M. J., Bhadbhade M., Messerle B. A. (2013). Organometallics.

[cit16] Kennedy D. F., Messerle B. A., Smith M. K. (2007). Eur. J. Inorg. Chem..

[cit17] Kennedy D. F., Messerle B. A., Rumble S. L. (20069). New J. Chem..

[cit18] Boudier A., Breuil P.-A. R., Magna L., Olivier-Bourbigou H., Braunstein P. (2012). J. Organomet. Chem..

[cit19] Kozlyuk N., Lopez T., Roth P., Acquaye J. H. (2015). Inorg. Chim. Acta.

[cit20] Kloskowski M., Pursche D., Hoffmann R.-D., Pöttgen R., Läge M., Hammerschmidt A., Glaser T., Krebs B. (2007). Z. Anorg. Allg. Chem..

[cit21] Okamura S., Maeda Y. (2003). J. Radioanal.
Nucl. Chem..

[cit22] García-Deibe A. M., Portela-García C., Fondo M., Mota A. J., Sanmartín-Matalobos J. (2012). Chem. Commun..

[cit23] Sanmartín-Matalobos J., Portela-García C., Fondo M., García-Deibe A. M. (2015). Cryst. Growth Des..

[cit24] Gerlach D., Brendler E., Heine T., Wagler J. (2007). Organometallics.

[cit25] Orr Jr L. B., Parsons E. J., Pennington W. T. (1992). Acta Crystallogr., Sect. C: Cryst. Struct. Commun..

[cit26] Bruker Topspin 3.2 (pl7), from Bruker BioSpin, Sun Microsystems Inc., 2010

[cit27] Bruker APEX2, SAINT and SADABS, Bruker AXS Inc., Madison, Wisconsin, USA, 2012

[cit28] Sheldrick G. M. (2015). Acta Crystallogr., Sect. C: Struct. Chem..

[cit29] Farrugia L. J. (2012). J. Appl. Crystallogr..

[cit30] Kabak M., Elmali A., Elerman Y. (1999). J. Mol. Struct..

[cit31] Akerman M. P., Chiazzari V. A. (2014). J. Mol. Struct..

[cit32] Pitt C. G., Bao Y., Thompson J., Wani M. C., Rosenkrantz H., Metterville J. (1986). J. Med. Chem..

[cit33] Bednar V., Elliott K. W., Byrd E., Woodford J. N. (2012). Chem. Phys. Lett..

[cit34] Frisch M. J. , Trucks G. W., Schlegel H. B., Scuseria G. E., Robb M. A., Cheeseman J. R., Scalmani G., Barone V., Mennucci B., Petersson G. A., Nakatsuji H., Caricato M., Li X., Hratchian H. P., Izmaylov A. F., Bloino J., Zheng G., Sonnenberg J. L., Hada M., Ehara M., Toyota K., Fukuda R., Hasegawa J., Ishida M., Nakajima T., Honda Y., Kitao O., Nakai H., Vreven T., Montgomery Jr. J. A., Peralta J. E., Ogliaro F., Bearpark M., Heyd J. J., Brothers E., Kudin K. N., Staroverov V. N., Kobayashi R., Normand J., Raghavachari K., Rendell A., Burant J. C., Iyengar S. S., Tomasi J., Cossi M., Rega N., Millam J. M., Klene M., Knox J. E., Cross J. B., Bakken V., Adamo C., Jaramillo J., Gomperts R., Stratmann R. E., Yazyev O., Austin A. J., Cammi R., Pomelli C., Ochterski J. W., Martin R. L., Morokuma K., Zakrzewski V. G., Voth G. A., Salvador P., Dannenberg J. J., Dapprich S., Daniels A. D., Farkas O., Foresman J. B., Ortiz J. V., Cioslowski J. and Fox D. J., Gaussian 09, Revision E. 01, Gaussian, Inc., Wallingford CT, 2009

[cit35] Dennington R. D. , Keith T. and Millam J. M., GaussView, Version 5, Semichem Inc., Shawnee Mission, KS, 2009

[cit36] Bauernschmitt R., Ahlrichs R. (1996). Chem. Phys. Lett..

[cit37] Casida M. E., Jamorski C., Casida K. C., Salahub D. R. (1998). J. Chem. Phys..

[cit38] Stratmann R. E., Scuseria G. E., Frisch M. J. (1998). J. Chem. Phys..

[cit39] Van Caillie C., Amos R. D. (1999). Chem. Phys. Lett..

[cit40] Van Caillie C., Amos R. D. (2000). Chem. Phys. Lett..

[cit41] Furche F., Ahlrichs R. (2002). J. Chem. Phys..

[cit42] Scalmani G., Frisch M. J., Mennucci B., Tomasi J., Cammi R., Barone V. (2006). J. Chem. Phys..

[cit43] Miertuš S., Scrocco E., Tomasi J. (1981). Chem. Phys..

[cit44] Miertus̃ S., Tomasi J. (1982). Chem. Phys..

[cit45] Macrae C. F., Bruno I. J., Chisholm J. A., Edgington P. R., McCabe P., Pidcock E., Rodriguez-Monge L., Taylor R., van de Streek J., Wood P. A. (2008). Mercury CSD 2.0. J. Appl. Crystallogr..

[cit46] Munro O. Q., Joubert S. D., Grimmer C. D. (2006). Chem. - Eur. J..

