# Supplementary material for: Self-assembled supramolecular structures of O,N,N′ tridentate imidazole–phenol Schiff base compounds
Source: RSC Adv. 2020 Feb 24;10(13):7867–78. doi: 10.1039/c9ra10488g (PMC9049879; doi:10.1039/c9ra10488g)
Supplement: RA-010-C9RA10488G-s001 [file RA-010-C9RA10488G-s001.pdf]

Electronic Supplementary Material (ESI) for RSC Advances.
This journal is © The Royal Society of Chemistry 2020

Self-assembled supramolecular structures of *O*,*N*,*N'* tridentate imidazole-phenol Schiff base compounds

Kristy-Lyn Barry,[a] Craig D. Grimmer,[a] Orde Q. Munro[b] and Matthew P. Akerman[a]*

[a]*School of Chemistry and Physics, University of KwaZulu-Natal, Private Bag X01, Scottsville, Pietermaritzburg, 3209, South Africa.*
[b]*Molecular Sciences Institute, School of Chemistry, WITS University, Johannesburg, South Africa.*

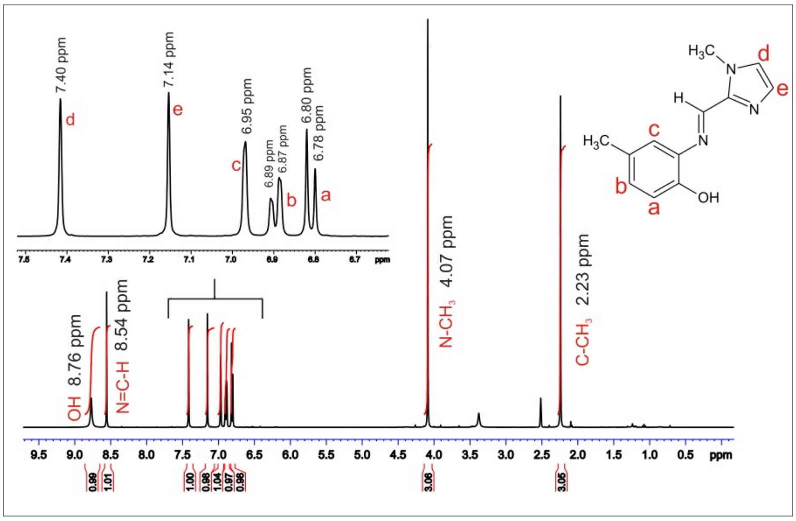

Figure S1: ^{1}H NMR spectrum of (1) in DMSO-d_6.

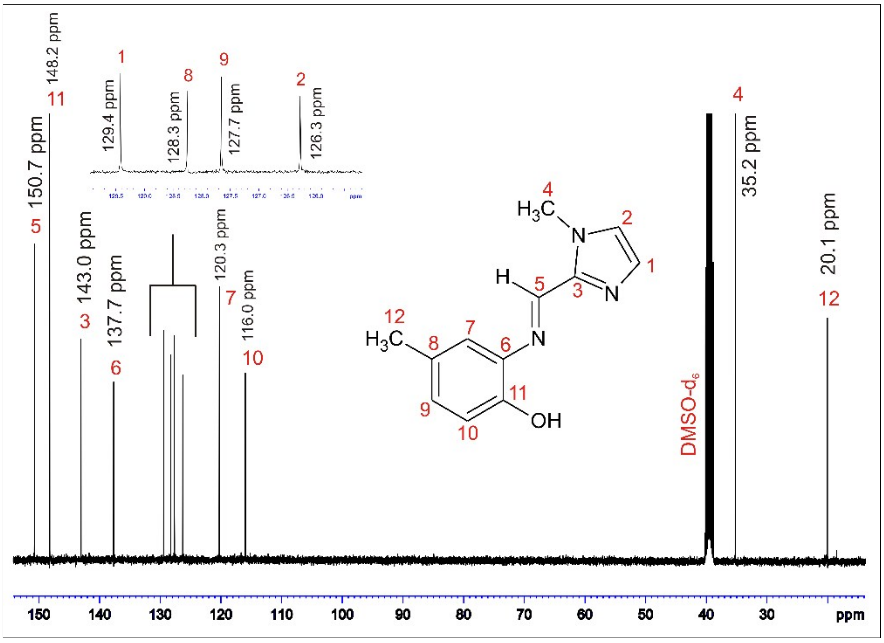

Figure S2: ^{13}C NMR spectrum of (1) in DMSO-d_6.

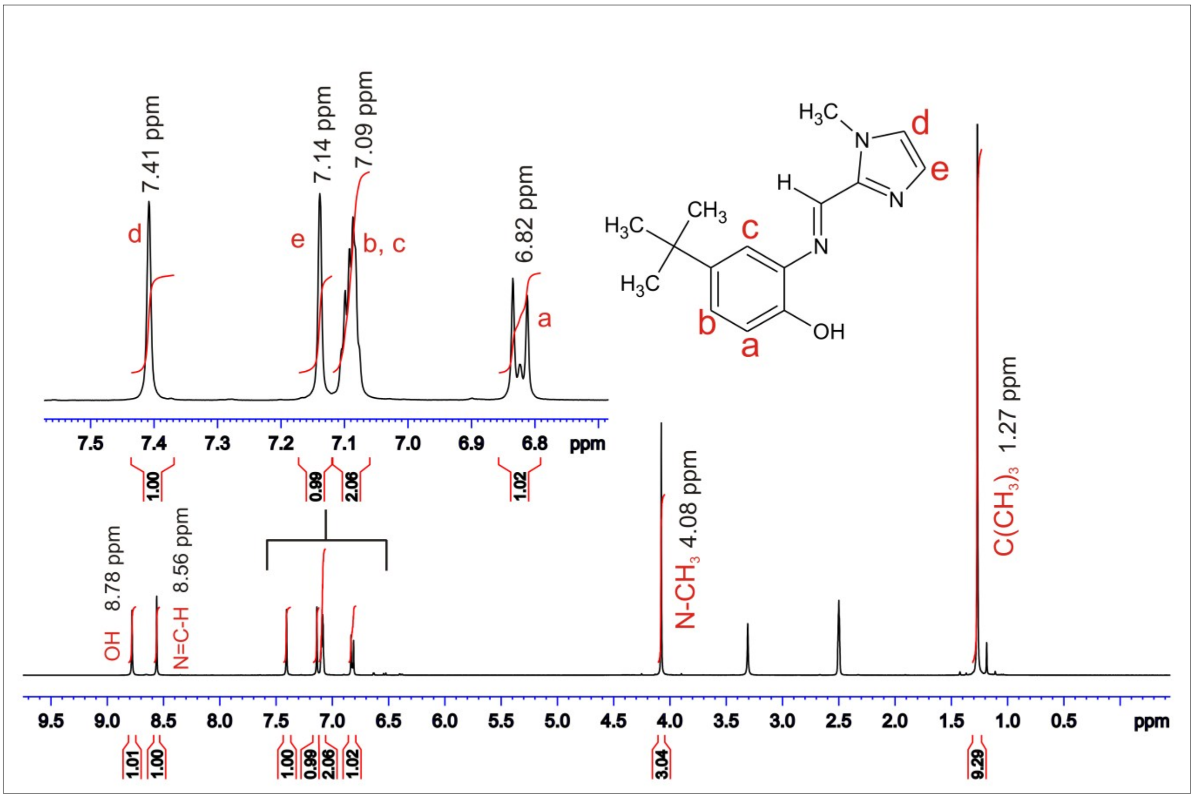

Figure S3: ^{1}H NMR spectrum of (2) in DMSO-d_6.

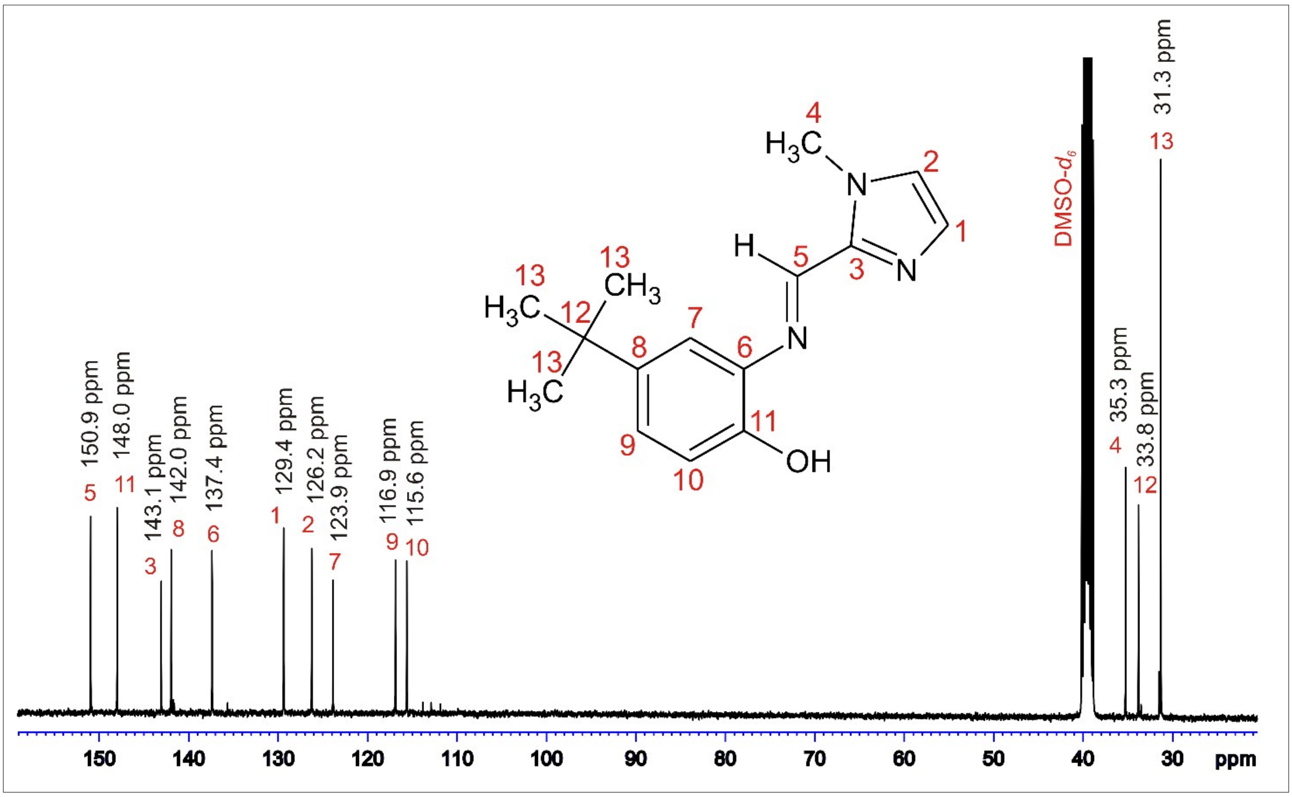

Figure S4: ^{13}C NMR spectrum of (2) in DMSO-d_6.

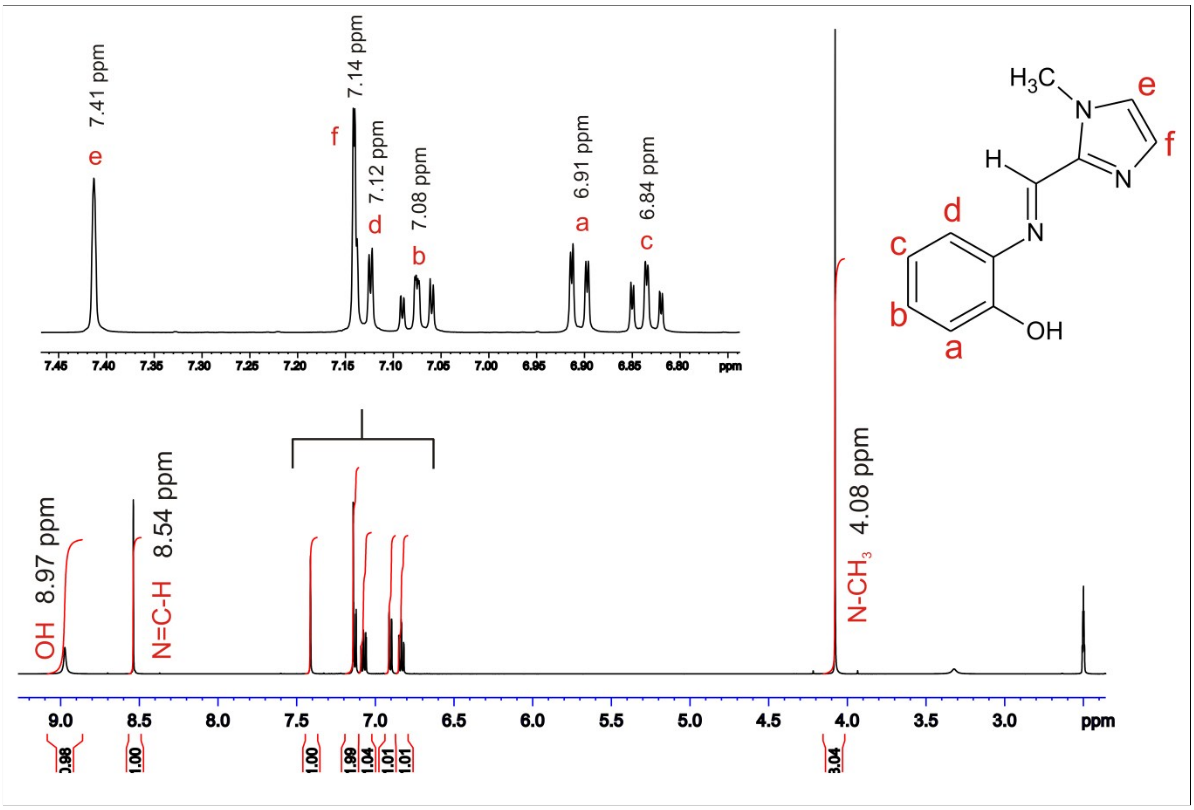

Figure S5: ^{1}H NMR spectrum of (3) in DMSO-d_6.

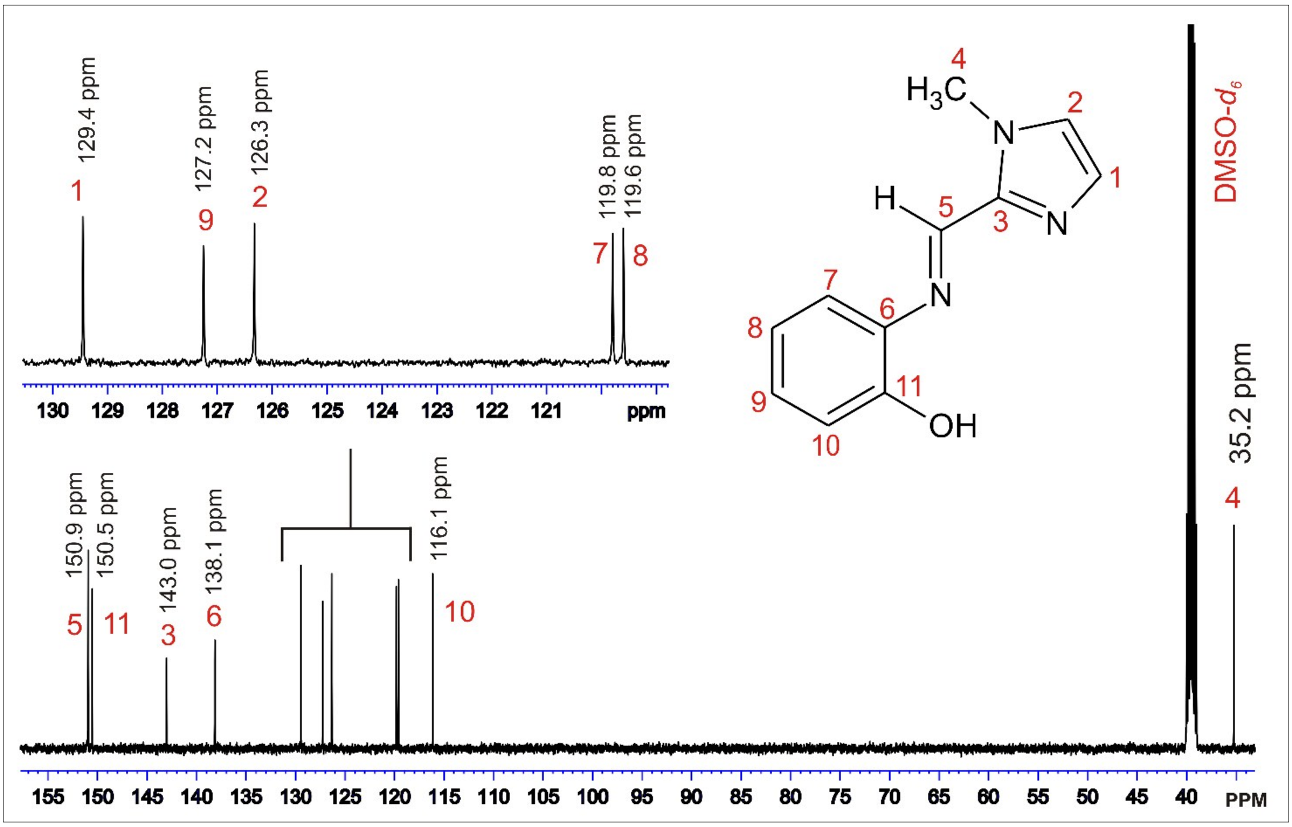

Figure S6: ^{13}C NMR spectrum of (3) in DMSO-d_6.

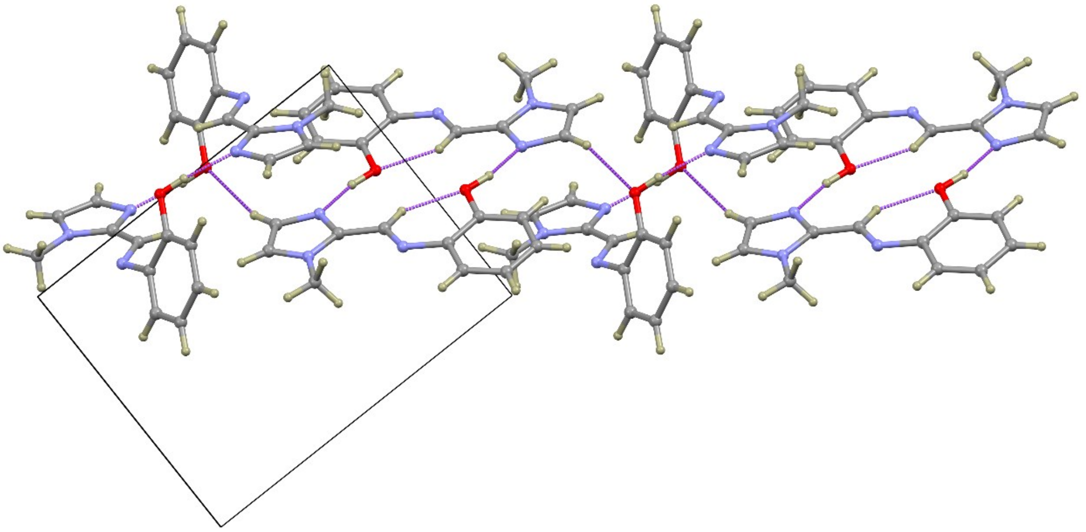

Figure S7: One-dimensional column of (3) viewed down the c-axis. All atoms are shown as spheres of arbitrary radius. The column comprises alternating dimers units of (3a) and (3b) linked by C–H···O interactions between the OH group and imidazole C–H.

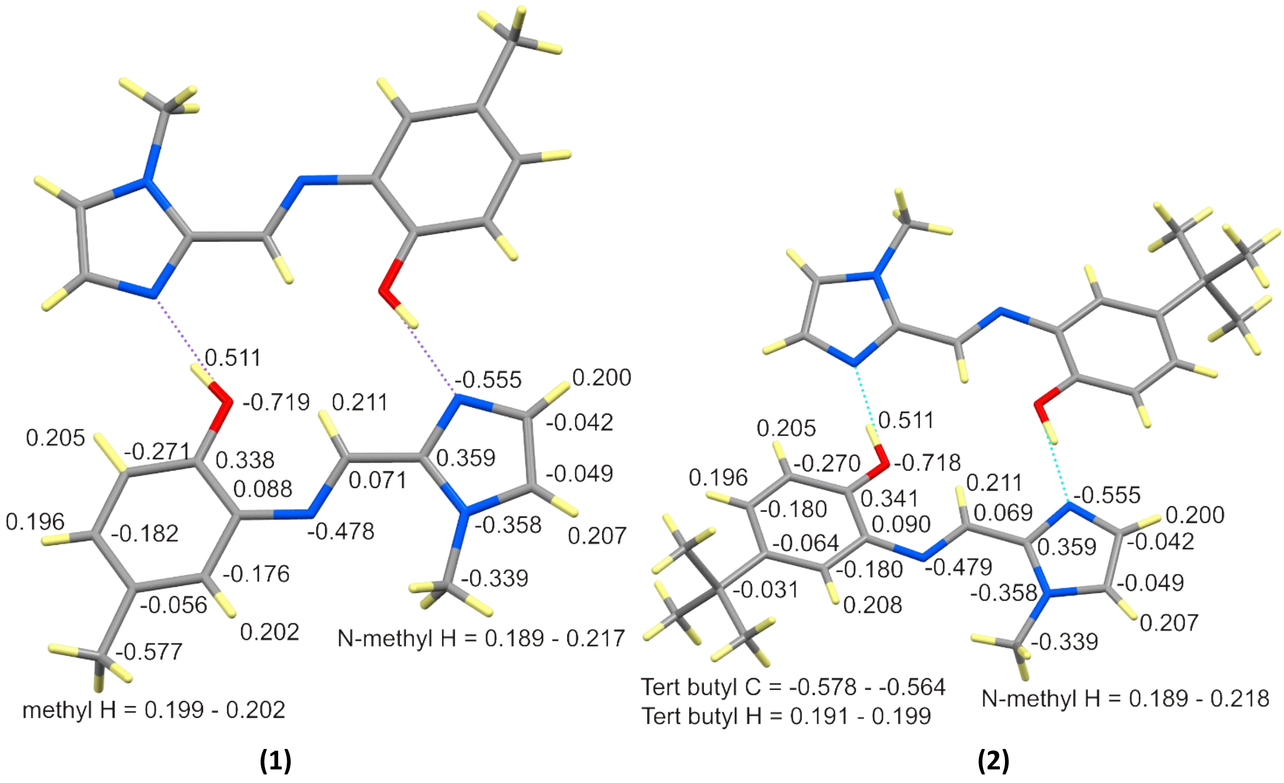

Figure S8: DFT calculated partial charges (e) for the dimeric structures of (1) and (2). The hydroxy proton and oxygen have the most positive and negative charges respectively.

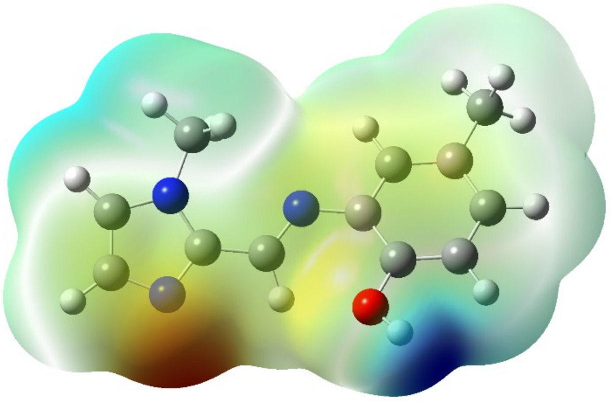

(1) MONOMER

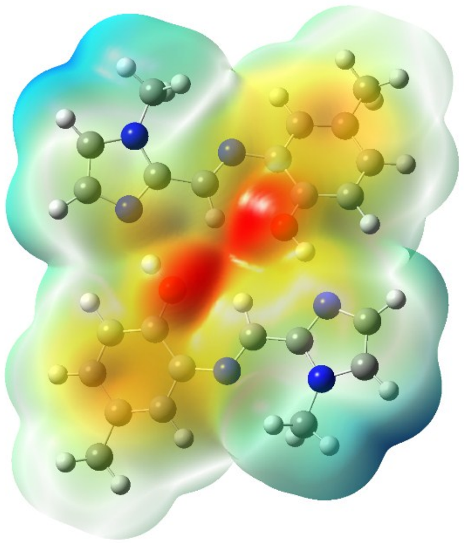

(1) DIMER

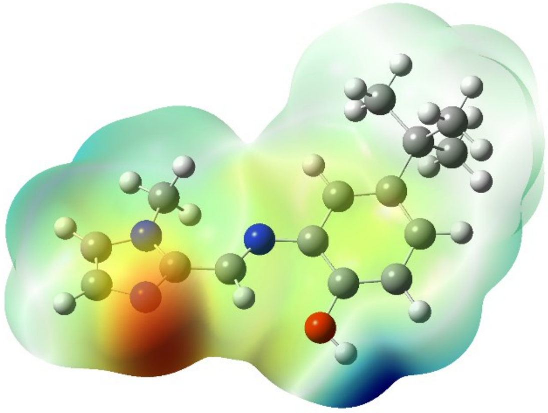

(2) MONOMER

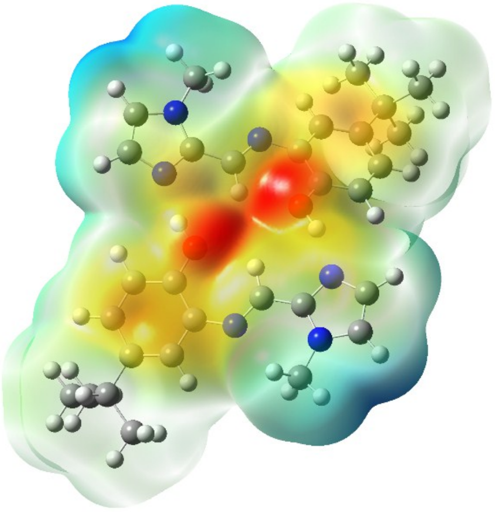

(2) DIMER

Fig. S9: Total SCF density molecular electrostatic potential maps of the monomer and dimer species of Ligands (1) and (2). Red regions indicate the most electron dense area and blue the most electropositive.

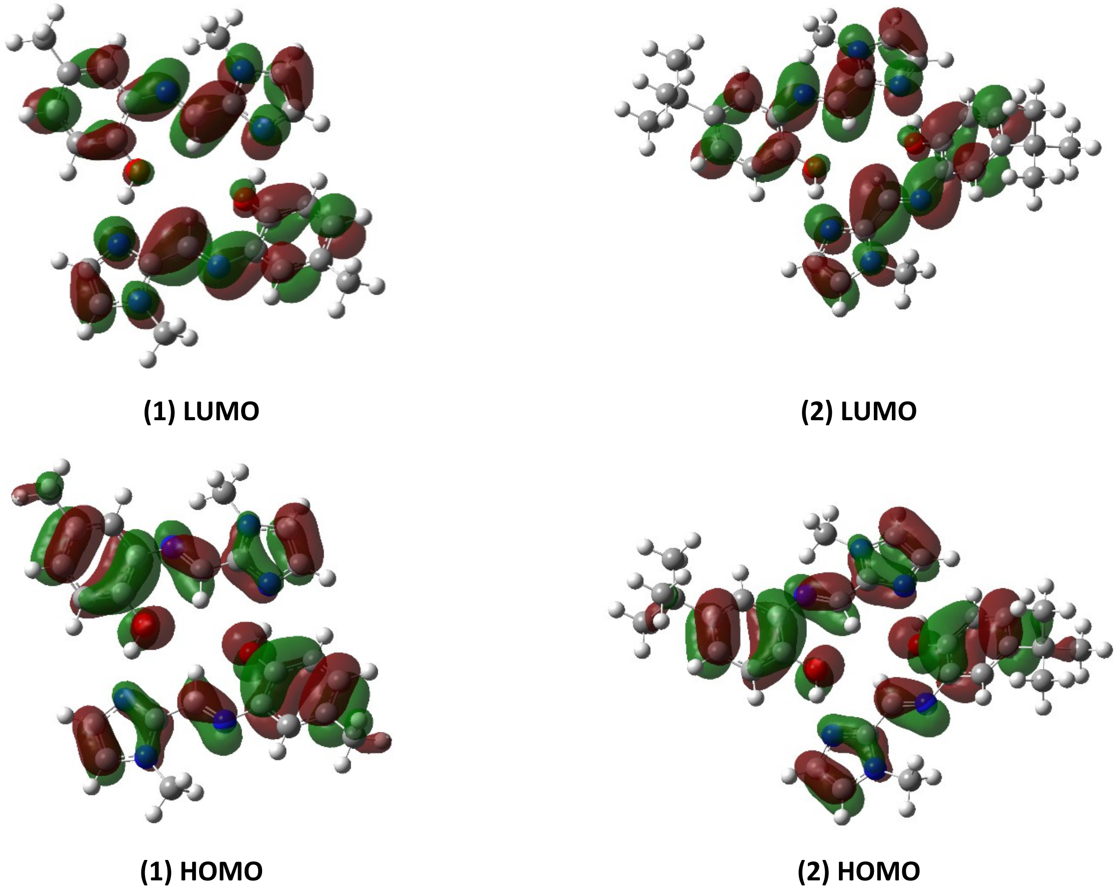

Figure S10: The HOMO and LUMO plots for geometry optimised dimer of (1) and (2) illustrating how the orbitals span both molecules.

Table S1: Summary of the major TD-DFT calculated electronic transitions for the monomeric and dimeric forms of (1) and the corresponding molecular orbitals (CH_3CN solvent continuum).

Wavelength (nm)[a]	Oscillator Strength, f	Molecular orbitals[b]	Contribution (%)[c]	Assignment[d]
**Monomer**				
346.80	0.5703	57 → 58	91	π(Imd, Im, Phenol, Me) → π*(Imd, Im, Phenol)
294.23	0.3213	54 → 58<br>55 → 58<br>56 → 58	11<br>45<br>32	π(N-Me, Imd, Im, Phen) → π*(Imd, Im, Phenol)<br>π(N-Me, Imd, Im, Phen) → π*(Imd, Im, Phenol)*<br>π(Imd, Im, Phenol, Me) → π*(Imd, Im, Phenol)
237.07	0.1371	54 → 58<br>55 → 58<br>57 → 59	36<br>12<br>34	π(N-Me, Imd, Im, Phen) → π*(Imd, Im, Phenol)<br>π(N-Me, Imd, Im, Phen) → π*(Imd, Im, Phenol)<br>π(Imd, Im, Phenol, Me) → π*(Phenol, Me)
**Dimer**				
356.28	0.6102	113 → 115<br>114 → 116	83<br>10	π(Imd, Im, Phenol, Me) → π*(Imd, Im, Phenol)<br>π(Imd, Im, Phenol, Me) → π*(Imd, Im, Phenol)
295.18	0.7446	109 → 116<br>110 → 115<br>112 → 115	16<br>20<br>45	π(Imd, Im, Phenol) → π*(Imd, Im, Phenol)<br>π(N-Me, Imd, Im, Phen) → π*(Imd, Im, Phenol)<br>π(Imd, Im, Phenol, Me) → π*(Imd, Im, Phenol)
238.57	0.2213	107 → 116<br>108 → 115<br>113 → 117<br>114 → 118	13<br>19<br>11<br>33	π(Imd, Im, Phenol) → π*(Imd, Im, Phenol)<br>π(Imd, Im, Phenol) → π*(Imd, Im, Phenol)<br>π(Imd, Im, Phenol, Me) → π*(Phenol)<br>π(Imd, Im, Phenol, Me) → π*(Phenol)

[a] Three of the 24 calculated excited states with the highest oscillator strengths are listed.

[b] For the monomer, the HOMO is orbital 57 and LUMO is 58; these are 114 and 115 for the dimer.

[c] Only orbitals with contributions >10% are listed.

[d] Imd = imidazole; Im = imine; Phenol = benzene ring + OH; Phen = benzene ring; N-Me = imidazole N-CH_3; Me = phenolic methyl substituent.
